# Recent extremes in Antarctic sea ice extent modulated by ocean heat ventilation

**DOI:** 10.1073/pnas.2530832123

**Published:** 2026-03-23

**Authors:** Earle A. Wilson, Lexi Arlen, Ethan C. Campbell

**Affiliations:** ^a^Department of Earth System Science, Stanford University, Stanford, CA 94305-4216; ^b^Polar Science Center, Applied Physics Laboratory, University of Washington, Seattle, WA 98105-6698

**Keywords:** Antarctic sea ice, Southern Ocean climate, Argo floats

## Abstract

Antarctic sea ice extent (SIE) has experienced unprecedented variability in recent decades, with record expansion through 2015, followed by an abrupt transition to sustained decline. Using over two decades of under-ice Argo float observations, we show that changes in ocean heat ventilation have modulated these extreme sea ice variations on interannual timescales. Between 2007 and 2015, the ocean thermocline warmed and shoaled within the Weddell Sea and off East Antarctica, with the former accounting for most of the interannual variability in Antarctic SIE. After 2016, as Antarctic SIE declined, surface salinity increased, enhancing exchange between the sharpened thermocline and surface waters. Idealized modeling of the Weddell Sea indicates that these upper ocean trends were due to concurrent variations in wind-driven Ekman upwelling and precipitation. During the sea ice expansion phase, increased precipitation enhanced ocean stratification, suppressing the upward flux of subsurface heat while promoting sea ice growth. However, between 2014 and 2016, a nearly three-fold increase in upwelling rates weakened the upper ocean stratification, releasing the accumulated subsurface heat. Though a similar sequence of events occurred along the East Antarctic margin, distinct upper-ocean trends and surface forcing in the Pacific sector of the Southern Ocean imply alternative drivers of recent sea ice loss in that region. Nevertheless, these results suggest that future multiyear Antarctic SIE variability will depend on the competing influences of wind-driven upwelling and surface freshwater fluxes.

## Significance

Antarctic sea ice is an integral component of the climate system, regulating heat and CO_2_ exchange between the surface and deep ocean. Contrary to the gradual ice loss predicted by climate models, we have observed ice expansion until 2015, followed by an abrupt and sustained decline in subsequent years. Using nearly two decades of under-ice Argo float data, we find that the ice expansion was partly due to surface freshening from enhanced precipitation that trapped subsurface ocean heat. After 2015, intensified wind-driven upwelling reversed freshening trends, releasing years of accumulated ocean heat that contributed to unprecedented sea ice loss. These results demonstrate the potential for wind-driven upwelling and freshwater fluxes to drive multiyear Antarctic sea ice trends.

**Fig. 1.**
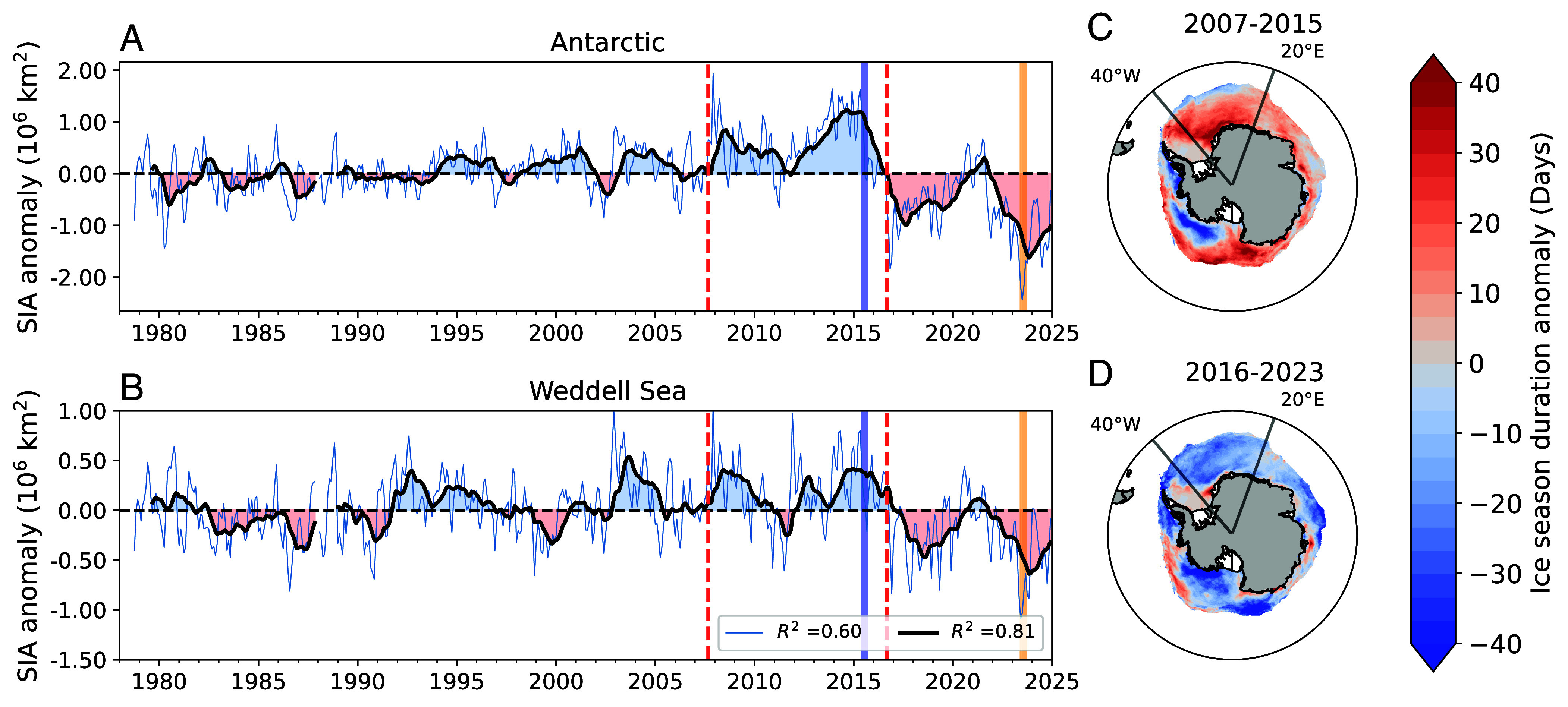
Southern Ocean sea ice trends. (*A* and *B*) Monthly sea ice area (SIA) for the Antarctic and Weddell Sea, between 40°W and 20°E. Anomalies are defined relative to the monthly climatology for 1980–2010. Black lines represent a 12-mo running mean. The dashed red lines highlight September 2007 and 2016, the starts of the accelerated sea ice expansion and decline periods as defined by ref. 8. The blue and orange vertical bars indicate the winter SIA maximum and minimum in 2015 and 2023, respectively. $R^{2}$ values in (*B*) indicate the fraction of variance in circumpolar SIA anomalies explained by corresponding anomalies in the Weddell Sea since 2007. (*C* and *D*) Sea ice season duration anomalies for 2007–2015 and 2016–2023 for regions where the climatological season duration is greater than 3 mo (*Materials and Methods*).

**Fig. 2.**
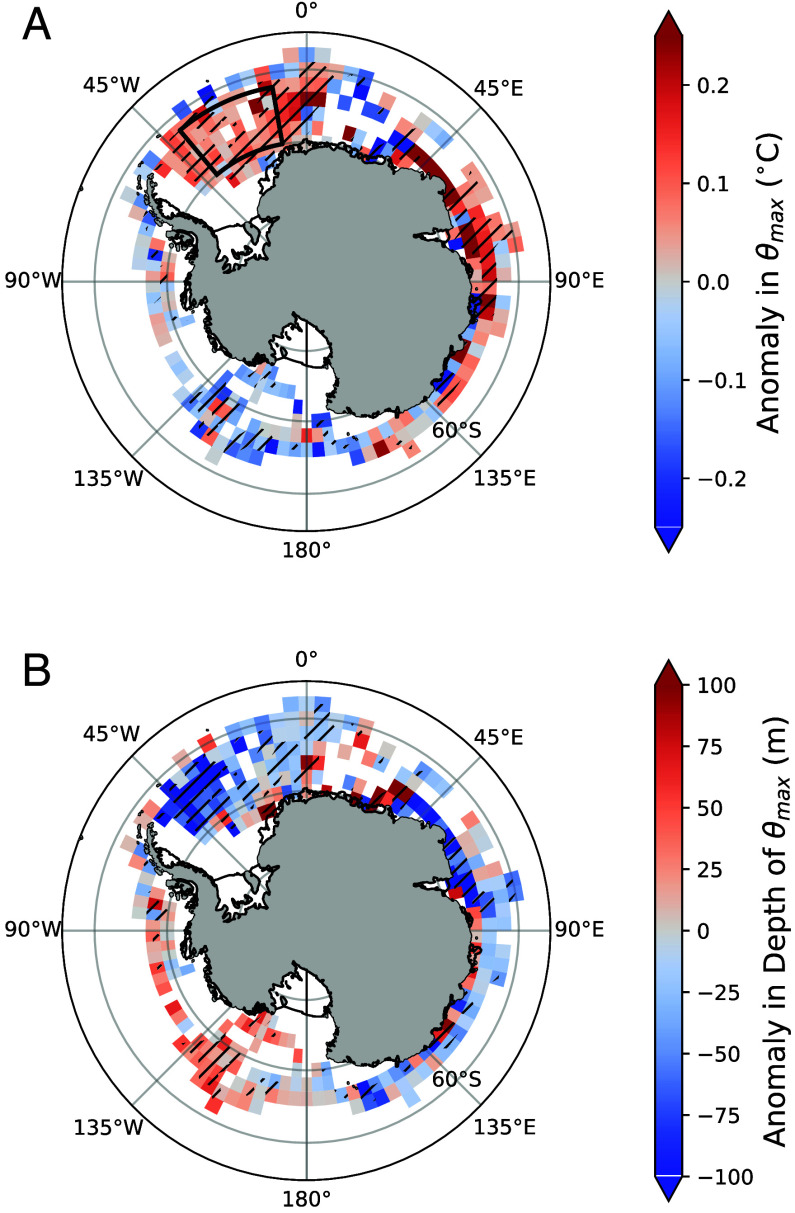
Changes in under-ice thermocline properties from the sea ice expansion period (September 2007–August 2016) to the retreat period (September 2016–December 2024), defined by vertical red lines in Fig. 1. (*A*) Difference in subsurface temperature maximum, $\theta_{\max}$, across the SIZ. The SIZ is identified here as regions where the wintertime mixed layer average temperature is less than −1 ^°^C. (*B*) As in (*A*) but showing changes in the depth of $\theta_{\max}$. Positive values indicate warmer and deeper $\theta_{\max}$ during the ice retreat period. Hatched regions denote grid cells where the differences are statistically significant at the 95% confidence level, based on 1,000 bootstrap resamples of the monthly averaged data.

Since the late 1970s, Antarctic sea ice extent (SIE) has generally expanded, reaching a record maximum in 2014 (Fig. 1; 1, 2). These trends abruptly reversed in 2016, when SIE declined to a record minimum and transitioned to a period of exceptionally low SIE (3–6). The persistence of this current low sea ice state raises the prospect of a lasting shift in Southern Ocean climate (7, 8). However, recent statistical analyses of Antarctic SIE trends demonstrate a marked increase in sea ice persistence since 2000 (9), suggesting an earlier structural shift in the Antarctic sea ice system. Here, we explore whether recent extremes in Antarctic sea ice expansion and decline share a common origin.

Past studies have mostly examined the sea ice expansion and retreat periods separately. For the ice expansion period, observational and modeling analyses have highlighted the strengthening of circumpolar westerly winds associated with the observed shift toward the positive phase of the Southern Annular Mode (SAM; 10, 11). Stronger eastward surface winds increase the northward Ekman transport of cool surface waters, which promotes sea ice expansion (12–14). These shifts in ocean advection were likely augmented by increases in precipitation and glacial meltwater fluxes, which strengthen upper ocean stratification and suppress the upward mixing of warmer deep water (15–17). Further, local changes in atmospheric circulation have been linked to remote modes of climate variability, notably the Interdecadal Pacific Oscillation (IPO; 18, 19). The abrupt reversal of Antarctic SIE trends in 2016 coincided with a dramatic weakening of the circumpolar westerly jet, triggered by atmospheric teleconnections originating from the tropical Indo-Pacific (20–24). However, the persistence of this sea ice decline suggests a lower-frequency forcing mechanism, possibly associated with a new ocean state. In situ observations show subsurface warming along the periphery of the seasonal sea ice zone (SIZ) leading up to and during the period of sea ice loss (8, 23). The appearance of unusually large open-ocean polynyas in the Weddell Sea in 2016 and 2017 further suggest a shift in ice–ocean interactions (25, 26).

Nevertheless, the role of upper ocean processes in driving recent Antarctic sea ice trends remains inconclusive. Observational analyses of in situ ocean data have been mostly limited to those collected north of 65°S (8, 23), omitting much of the SIZ. Additionally, a consistent theory is lacking for how shifts in the ocean state may facilitate such an abrupt transition between maximum and minimum SIE. One leading hypothesis suggests a two-timescale surface temperature response following a shift toward positive SAM, whereby the strengthening of the circumpolar westerlies initially causes surface cooling and sea ice expansion, due to increased northward Ekman transport, that eventually reverses due to enhanced upwelling CDW (13). However, these temperature responses are poorly constrained, with some climate models showing surface warming within a few months after an increase in SAM while others exhibit no warming after several decades (14, 27–30).

Though some ocean general circulation models forced with observed atmospheric conditions can approximately reproduce recent Antarctic SIE trends, diagnosing the relative importance of ocean processes remains challenging. Some model analyses highlight the role of deep ocean heat ventilation in sustaining recent sea ice decline (31, 32), while others implicate surface processes, such as enhanced ocean warming near the ice edge and anomalous atmospheric heat transport (33, 34). These apparent disagreements may stem from differences in ocean initialization, which is difficult to constrain with sparse observations. Additionally, the relative importance of oceanic and atmospheric processes are difficult to disentangle since ocean-driven sea ice anomalies will necessarily leave an imprint on surface heat and momentum fluxes.

Here, we build on previous observational analyses by utilizing over two decades of under-ice Argo float profiles to characterize the spatiotemporal changes in upper ocean heat content, stratification, and mixed layer properties across the Southern Ocean SIZ. Although these in situ observations are relatively sparse, they provide essential ground truth, as even data-assimilating ocean models can produce divergent representations of the ocean’s state (35). Using an idealized sea ice–ocean model, we evaluate potential forcing mechanisms that may explain upper ocean trends and their impact on sea ice trends. A key conclusion is that recent decadal Antarctic sea ice trends have likely been modulated by the ventilation of subsurface ocean heat.

## Results

### Trends in Antarctic Sea Ice Area and Seasonality.

Much of the circumpolar variability in Antarctic sea ice area (SIA) over the past two decades can be attributed to changes in the Weddell Sea (Fig. 1 *A* and *B*). Since 2007, monthly averaged SIA in the Weddell Sea has accounted for over 60% of the variability in corresponding circumpolar trends—the variance explained by the Weddell Sea increases to over 80% when both time series are smoothed with a 12-mo running mean. Given the Weddell Sea’s predominant role in driving circumpolar sea ice variability and its relatively high density of in situ measurements (*SI Appendix*, Figs. S1 and S9), we focus on this region while recognizing that its trends may reflect circumpolar-scale forcing rather than local processes alone. Further, while 2007–2015 and 2016–present are statistically distinct periods of anomalous circumpolar sea ice expansion and retreat, respectively (8), these categorizations may not apply to regional sea ice trends. Antarctic SIA anomalies also correspond to pronounced shifts in sea ice seasonality (Fig. 1 *C* and *D*). During 2007–2015, the duration of ice cover in the Weddell Sea was one month longer than climatology, with the largest increases along the continental slope. For the ice expansion period, the Pacific sector exhibited a dipole pattern of shorter season length in the Amundsen and Bellingshausen Seas and longer sea ice duration in the western Ross Sea, which mirrors the well-documented sea ice response to the strengthening of the Amundsen Sea Low (36). During the retreat period, season length decreased across most sectors with similar magnitudes, as previously reported (37).

### Observed Upper Ocean Anomalies in the SIZ.

We next assess in situ, under-ice Argo observations during the circumpolar sea ice expansion and retreat periods. Water column properties were first computed for individual profiles and then averaged monthly within $4^{{}^{\circ}}$ longitude by $2^{{}^{\circ}}$ latitude bins (*Materials and Methods*). We use the subsurface potential temperature maximum ($\theta_{\max}$), located at the base of the main thermocline, to characterize the variability of CDW (hereafter used to denote all regional variants of this water mass) across the SIZ. Unlike near-surface properties, $\theta_{\max}$ shows little seasonality, allowing us to aggregate data across all months without concern for seasonal aliasing. Further, vertical temperature gradients immediately below the winter mixed layer are sharpest where $\theta_{\max}$ is shallow and warm (*SI Appendix*, Figs. S2 and S3). Thus, $\theta_{\max}$ is a convenient measure of heat availability below the mixed layer.

The Argo float measurements reveal that, across the Weddell Sea and the East Antarctic margin, the under-ice thermocline was substantially warmer and shallower during the sea ice retreat period compared to the preceding sea ice expansion period (Fig. 2 and *SI Appendix*, Fig. S2). In the Weddell Sea, $\theta_{\max}$ was approximately 0.1 °C warmer and up to 100 m shallower during the ice retreat period, with the largest differences occurring in the western Weddell Sea. The subsurface warming is even more striking along the East Antarctic coastline, where $\theta_{\max}$ increased by more than 0.1 °C, consistent with the reported poleward shift of CDW in the region (38, 39). In the Pacific sector, the thermocline generally cooled and deepened during the sea ice retreat period.

During winter months (June–September), when the mixed layer is in direct contact with the thermocline, there is a coherent warming pattern within and immediately below the mixed layer in the Weddell Sea for the sea ice retreat period (*SI Appendix*, Figs. S3 and S4). Outside the Weddell Sea, where winter data are more sparse, no coherent anomalies in sub-mixed-layer gradients are observed, except across the northern Ross Sea, between 135°W and 135°E, where the winter mixed layer was consistently warmer, saltier, and deeper during the sea ice retreat period (*SI Appendix*, Fig. S4).

### Temporal Variations in Upper Ocean Properties in the Weddell Sea.

**Fig. 3.**
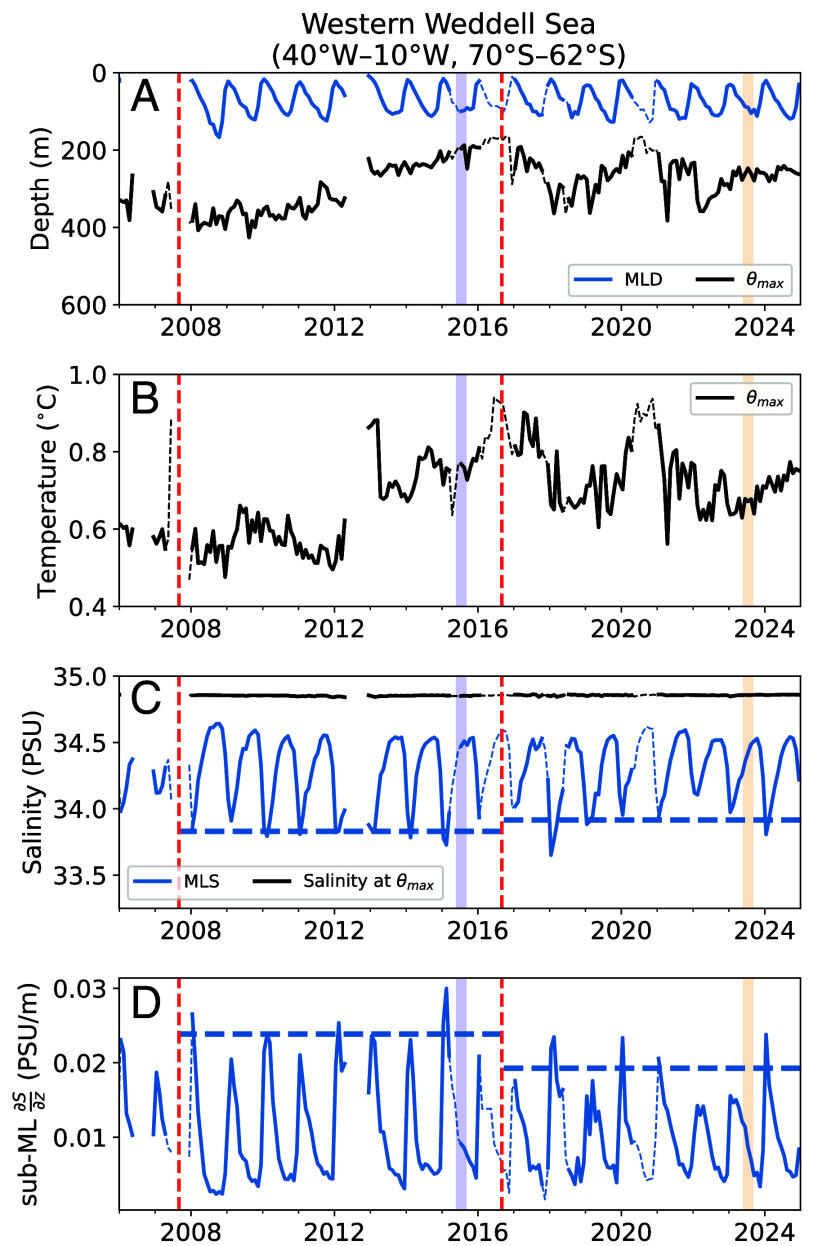
Monthly averaged upper-ocean properties in the western Weddell Sea (defined by the box in Fig. 2*A*) for years 2006-2024. (*A*) Mixed layer depth (MLD) and depth of $\theta_{\max}$. (*B*) $\theta_{\max}$. (*C*) Mixed layer salinity (MLS) and salinity at the depth of $\theta_{\max}$. (*D*) Salinity gradient below the mixed layer (ML). In panel (*C*), the dashed horizontal lines show the average annual minimum salinity over the sea ice advance and retreat periods; in panel (*D*), the horizontal lines represent the average annual maximum vertical salinity gradient for each period. Thin dashed curves represent monthly averages using all available data while thick solid curves highlight months with more than 10 profiles. Vertical lines demarcate the starts of the sea ice advance and retreat periods and the winter SIA maximum and minimum, as in Fig. 1.

While $\theta_{\max}$ anomalies in the Weddell Sea and along the East Antarctic margin lend support to the hypothesis that the recent decline in Antarctic sea ice has been sustained by elevated ocean heat fluxes, the interannual variations in upper ocean properties offer a more nuanced perspective. We first focus on the western Weddell Sea between 40°W to 10°W and 70°S to 62°S (box in Fig. 2*A*), which features relatively homogeneous upper ocean properties and temporally consistent data coverage (*SI Appendix*, Figs. S1 and S2). We deliberately avoid the Maud Rise seamount, near 0°E and 65°S, due to its distinct water mass properties and local dynamics. In the western Weddell Sea, $\theta_{\max}$ steadily shoaled from an average depth of ∼400 m between 2008 and 2010 to almost 200 m in 2016 (Fig. 3*A*). After 2016, $\theta_{\max}$ deepened before rebounding in 2018, eventually settling at an average depth of ∼300 m. These variations are also reflected in $\theta_{\max}$ itself, which warmed from 0.5 °C to 0.9 °C between 2008 and 2016 (Fig. 3*B*). This increase in $\theta_{\max}$ indicates that the thermocline shoaling was influenced by an increase in CDW inflow or reduced heat ventilation, rather than the mere vertical displacement of isopycnals. More importantly, the Weddell Sea subsurface warming trend began several years before the observed decline in sea ice area. In fact, the thermocline was warmer and shallower during the winter of 2015, when SIE was near its record maximum, than in the winter of 2023 when sea ice reached a record low (blue and orange vertical bars in Fig. 3).

The apparent inconsistency between the thermocline and SIE trends may be reconciled by covariations in surface salinity. The near-surface layers of the Weddell Sea upper ocean were generally fresher during the sea ice expansion period compared to the ice retreat period, especially during summer months (Fig. 3 *C* and *D*). A lower surface salinity strengthens the stratification and reduces upward mixing of heat. Between 2008 and 2015, minimum summertime mixed layer salinity (MLS) typically reached as low as 33.8 PSU—after 2016, summer MLS rarely decreased below 34 PSU (Fig. 3*C*). These results are consistent with a recent satellite-based surface salinity retrieval (40). The summer halocline, characterized by sub-mixed-layer salinity gradients, shows a similarly dramatic weakening after 2015. Even though salinity increased within the main thermocline, the near-surface increases were several times larger (*SI Appendix*, Figs. S2*I* and S4*F*). Therefore, upper ocean stratification was weaker overall during the sea ice retreat period. Crucially, the sharp reversal in halocline strength in 2015–2016 coincided with maximal thermocline warming and shoaling, suggesting heightened availability of warm, salty CDW to the surface.

Nevertheless, since sea ice directly influences surface salinity, the direction of causality between these changes is not readily apparent from the Argo float data alone. If sea ice were thicker during the ice expansion period, for example, enhanced sea ice melt could strengthen stratification during the summer, whereas the opposite might occur during the ice retreat period. However, as shown in the following sections, the shift in MLS can be explained by surface forcing processes that are mostly independent of sea ice variations.

Similar thermocline and upper ocean salinity trends are observed along East Antarctica (*SI Appendix*, Figs. S5 and S6), albeit with thermocline shoaling less pronounced in the eastern Indian Ocean sector, further away from the Weddell Sea. In the Ross Sea, the limited data prior to 2016 show no discernible shift in thermocline properties in the years leading up to the sea ice loss period (*SI Appendix*, Fig. S7). Thermocline trends were similarly muted in the Bellingshausen and Amundsen Seas over the past two decades (*SI Appendix*, Fig. S8).

### Surface Stress and Freshwater Flux Trends in the Weddell Sea.

**Fig. 4.**
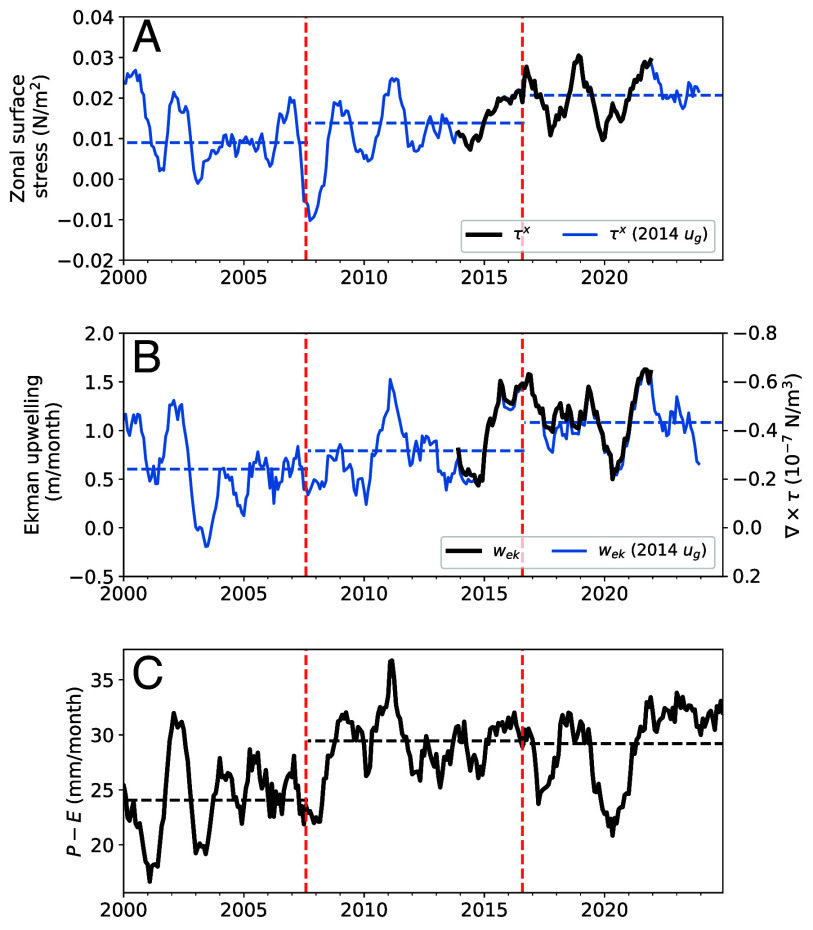
Surface stress and freshwater flux trends in the western Weddell Sea (defined by the box in Fig. 2*A*) for 2000–2024. (*A*) Eastward component of the surface ocean stress. (*B*) Ekman upwelling rate and equivalently the curl of the surface ocean stress. (*C*) Precipitation minus evaporation, P−E. In (*A* and *B*), the blue lines show an extended record calculated using repeating surface geostrophic velocities from 2014. Plots have been smoothed using 12-mo running averages. The dashed horizontal lines show the temporal means of the surface stresses (assuming geostrophic velocities from 2014) and P−E for the three periods: January 2000 to August 2007, September 2007 to August 2016, and September 2016 to December 2024, which are demarcated by the vertical dashed lines.

The trends in Weddell Sea upper ocean stratification and thermocline properties coincided with anomalous Ekman upwelling and surface freshwater fluxes (Fig. 4). Ekman upwelling is proportional to the curl of the surface ocean stress τ (Fig. 4*A* depicts its eastward component), defined by the sum of the air–ocean ($\tau_{a}$) and ice–ocean ($\tau_{i}$) stresses (*Materials and Methods* and *SI Appendix*, Fig. S10). $\tau_{i}$ depends on the difference between sea ice and surface ocean velocities, where the latter is given by the sum of Ekman and geostrophic currents. Surface geostrophic flow is estimated from satellite-based dynamic ocean topography, available between 2013 and 2021.

Between 2014 and 2016, Ekman upwelling across the central Weddell Sea (box in Fig. 2*A*) increased from approximately 0.5 m/mo to almost three times that rate in 2016 (Fig. 4*B*). Upwelling remained relatively high over the next five years, with an average rate of ∼1 m/mo. To contextualize this short record of variability, we also compute surface stresses using repeating surface ocean geostrophic velocities from 2014, which provide surface stress estimates for the past several decades (blue curves in Fig. 4 and *SI Appendix*, Fig. S10). When these surface stress estimates overlap, they are highly similar, indicating that geostrophic velocities play a minor role in driving interannual surface stress fluctuations in this region. The longer surface stress record shows a decadal increase in zonal surface stress and upwelling extending from the early 2000s through to the sea ice retreat period (Fig. 4 *A* and *B*). Further, by fixing sea ice concentration (SIC) to 2014 conditions, we find the subsequent reductions in sea ice cover have increased upwelling rates by 10 to 20% (*SI Appendix*, Fig. S10*C*). More generally, however, interannual variations in surface stress and upwelling rates largely correspond to fluctuations in air–sea stresses, $\tau_{a}$.

Similar to the surface stress trends, a gradual decadal increase in net air–sea freshwater fluxes has occurred over the Weddell Sea, ranging from an average of 24 mm/mo in the early 2000s to 30 mm/mo since 2007 (Fig. 4*C*). Though precipitation minus evaporation (P−E) exhibited large fluctuations after 2016, air–sea freshwater fluxes have remained generally higher than the long-term average, especially since 2021. These Weddell Sea P−E trends mirror broader patterns across the Atlantic sector of the Antarctic SIZ (*SI Appendix*, Fig. S11*F*). Thus, even though sea ice intercepts and redistributes snow across regions (41, 42), these P−E trends reflect basin-scale increases in air–sea freshwater fluxes across the Weddell Sea region.

Likewise, there are decadal increases in zonal surface stress and Ekman upwelling across the broader Antarctic SIZ (*SI Appendix*, Fig. S11). On interannual time scales, the surface stress and precipitation forcing in the Weddell Sea strongly covary with those along East Antarctica, particularly in the Cooperation Sea region between 45°E and 90°E (*SI Appendix*, Fig. S11). The surface forcing in the Weddell and Bellingshausen Seas is similarly in sync on interannual time scales. However, there is minimal covariance in surface forcing between the Ross and Weddell Seas, even though both regions exhibit similar decadal trends.

### The Impact of Ekman Upwelling and *P* − *E* on Sea Ice Trends.

To isolate the relative influence of upwelling and freshwater flux variability on sea ice trends, we conducted perturbation experiments using an idealized, 1D sea ice–ocean model (*Materials and Methods*). Here, the intent is to constrain the impact of these forcings on interannual sea ice and upper ocean variability, rather than provide a comprehensive evaluation of all relevant processes. As before, we focus on the SIZ in the western Weddell Sea. An ensemble of simulations were conducted, each initialized with one of 40 randomly selected summertime temperature and salinity profiles from the western Weddell Sea (*SI Appendix*, *Methods* and Fig. S13). Using a control ensemble equilibrated with repeated surface forcing from the year 2000 (*SI Appendix*, Figs. S14 and S15), we tested the isolated and combined effects of subsequent variations in upwelling and freshwater fluxes.

**Fig. 5.**
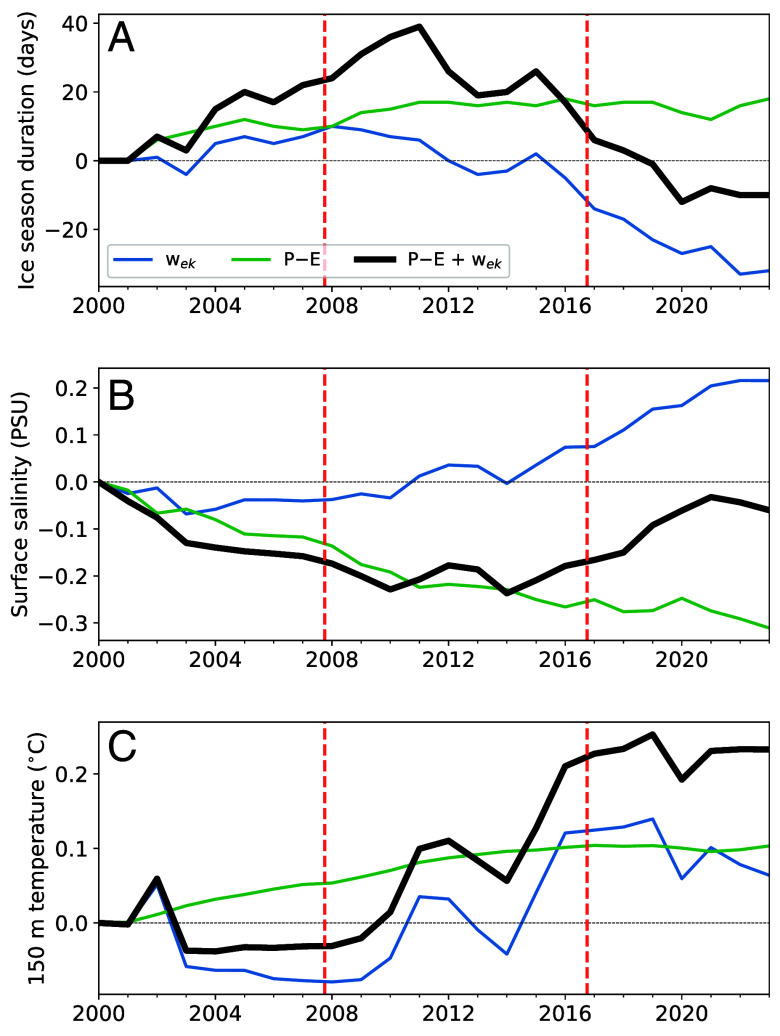
1D ice–ocean model simulations of upper ocean and sea ice properties forced with observed variations in upwelling and precipitation across the Weddell Sea for years 2000-2024. (*A*) Annual-mean ice season duration anomalies when the model is forced individually with interannually varying Ekman upwelling (blue), P−E (green), and both (black). (*B* and *C*) Like (*A*) but for ocean surface salinity and temperature at 150 m, respectively. Perturbation runs were branched from a control simulation equilibrated to surface forcing from the year 2000 (*SI Appendix*, Figs. S14 and S15). Each line represents the median of a 40-member ensemble initialized with different summertime Argo float profiles from the Weddell Sea. Dashed vertical lines indicate the starts of the sea ice advance and retreat periods.

When forced only with interannually varying $w_{\mathrm{ek}}$, the model simulates, on average, a slight increase in sea ice duration and thickness up until 2008, followed by a more pronounced decline thereafter (blue line in Fig. 5*A* and *SI Appendix*, Fig. S16). The latter sea ice reduction is accompanied by an increase in surface salinity and thermocline temperature, a response consistent with the positive Ekman upwelling trend during this period (Fig. 4*B*). The sea ice decline also coincides with increases in the winter mixed layer depth and ocean-ice heat fluxes (*SI Appendix*, Fig. S17), indicating enhanced entrainment of thermocline heat into the surface layer. When the P−E forcing is applied in isolation, the model produces sea ice growth, surface freshening, mixed layer shoaling, reduced ocean-ice heat fluxes, and thermocline warming during the entire perturbation period (2000–2023)—consistent with the positive trends in air–sea freshwater fluxes (Fig. 4*C*).

When $w_{\mathrm{ek}}$ and P−E trends are applied simultaneously, sea ice duration increases until 2010, with a median maximum gain of ∼40 d, and generally declines afterward, reaching a minimum ice duration anomaly of approximately −10 d after 2020. The reversal of sea ice durations mirror those of wintertime ocean-ice heat fluxes (*SI Appendix*, Fig. S17). These ice duration anomalies are similar in magnitude to observed anomalies for the same periods (Fig. 1 *C* and *D*). Individual ensemble members exhibit more extreme sea ice responses, with some producing perennial sea ice between 2008 and 2016 (*SI Appendix*, Fig. S16). This wide spectrum in the ice response reflects the spread in upper ocean properties across our Weddell Sea focus region, which adjoins the perennial sea ice pack along the Antarctic Peninsula. Thus, the more extreme sea ice growth response may plausibly reflect a slight expansion of the perennial ice pack between 2008 and 2016. Surface salinity mirrors the trends in sea ice, with the surface being relatively fresh during sea ice growth and saltier while sea ice declines. Notably, thermocline temperatures increase throughout both the ice thickening and thinning periods. Further, there is relatively small ensemble spread in the upper ocean response.

Additional perturbation simulations using interannually varying surface stresses show relatively small impacts on upper ocean and sea ice trends (*SI Appendix*, Figs. S16 and S17). This suggests that despite the increases in surface stress, wind-driven entrainment played a minor role in recent sea ice loss in the Weddell Sea. However, the daily averaged surface stresses, which are limited by the temporal resolution of sea ice and surface geostrophic velocities, likely underestimate the impact of wind-driven mixing associated with subdaily synoptic weather events, such as storms. Therefore, these results do not conclusively rule out wind-driven mixing as a major contributing factor.

Given the simplicity of the model, we should not expect a close correspondence with observations. Yet, the simulations with the combined effects of $w_{\mathrm{ek}}$ and P−E qualitatively capture key observed interannual variations, in particular: i) the positive sea ice anomalies between 2007 and 2016 and mostly negative anomalies thereafter, ii) the relatively low surface salinity during the ice expansion period and subsequent increase during the ice decline phase, and iii) the general increase in thermocline temperatures after 2008. These similarities are particularly remarkable considering the simulations were not forced with observed air–sea heat fluxes and do not account for lateral advection. Still, there are important discrepancies with observations, such as the underestimation of positive sea ice anomalies in 2014–2015 and the rate and relative magnitude of the sea ice decline after 2016. The latter is consistent with past studies attributing the sea ice loss during that year to atmospheric circulation anomalies (21, 23, 24). Nevertheless, these results strongly imply that upper ocean thermodynamics have substantially modulated Antarctic sea ice trends on multiyear timescales.

## Discussion

Under-ice Argo float data reveal that Antarctic sea ice has been increasingly exposed to warm CDW over the past two decades. While our results support earlier inferences that subsurface warming has sustained recent Antarctic sea ice decline (7, 8, 23, 31, 37), we illuminate the causal chain of events by emphasizing the critical role of surface salinity and stratification trends. We further highlight the regional dependence of these processes, with thermocline warming and shoaling being most apparent in the Weddell Sea and offshore of East Antarctica. In contrast, the thermocline in the Pacific sector of the Antarctic SIZ has mostly cooled and deepened since 2007. Within the Weddell Sea, the halocline was relatively strong during the 2007–2015 sea ice expansion period, which suppressed vertical heat fluxes and contributed to the concurrent subsurface warming. The reduction in halocline strength after 2015 coincided with subsurface cooling, implying greater heat flux to the surface that would suppress sea ice growth.

Idealized modeling of the Weddell Sea ice-ocean system suggests that air–sea freshwater fluxes and wind-driven upwelling modulated decadal variations in sea ice and upper ocean stratification. Since the early 2000s, upwelling rates have generally increased in the Weddell Sea, enhancing heat and salinity fluxes to the ocean surface. While upwelled heat can be efficiently ventilated to the overlying atmosphere or sea ice, the accompanying salt accumulates in the mixed layer, reducing stratification and amplifying the vertical mixing of warm, salty subsurface waters—a positive feedback. However, regional upwelling trends alone would have likely triggered negative sea ice anomalies several years earlier than observed. Instead, relatively high net precipitation helped to stabilize the water column, reduce vertical heat fluxes, and promote sea ice growth. The destabilizing effect of upwelling eventually prevailed when Ekman upwelling rapidly increased between 2014 and 2016, reversing surface freshening trends and facilitating the release of accumulated subsurface heat.

Although a similar mechanism likely contributed to recent sea ice loss along the East Antarctic margin, other processes appear to have been more influential in the Pacific sector, where interannual upper-ocean and surface forcing trends differ substantially. The Bellingshausen and Amundsen Seas stand out as having similar precipitation and upwelling trends and variability as the Weddell Sea (*SI Appendix*, Figs. S11 and S12), but no clear thermocline warming or shoaling trend over the past two decades. The contrasting ocean response may reflect differences in local ocean dynamics. Notably, the ocean circulation across the Ross, Bellingshausen, and Amundsen Seas is strongly coupled (43, 44), raising the likelihood that thermocline anomalies in the latter regions are influenced by nonlocal processes. Furthermore, weaker stratification in the Weddell Sea and along the East Antarctic margin enables relatively high vertical heat fluxes (45), making sea ice growth in these regions more sensitive to subsurface heat ventilation than in the Pacific sector. A similar regional contrast in the ocean’s thermodynamic control on sea ice variability may have existed during the sharp decline in Antarctic sea ice extent in the late 1970s (46).

While both the periods of accelerated Antarctic sea ice expansion and retreat are consistent with vertical upper ocean thermodynamics, these results do not preclude the importance of other processes in driving sea ice variations. The increase in wind stress curl leading up to 2016 also strengthened the Weddell Gyre (35, 47), which increases the inflow of CDW and the heat content of the water column (48). Further, changes in surface stresses and gyre circulation likely impacted the magnitude and distribution of sea ice freshwater fluxes, which are climatologically similar in magnitude to P−E fluxes and have a leading order impact on the spatial patterns of surface salinity (42). Additionally, atmospheric circulation anomalies played a significant role in the pronounced sea ice decline in 2016 and subsequent periods (21, 23, 24, 49). Finally, the circumpolar increase in surface stress may have also enhanced the upward turbulent mixing of warm thermocline waters and the mechanical breakup of sea ice, particularly near the ice edge. Taken together, our results suggest that upwelling and precipitation trends acted in concert with other processes to drive the extreme variability in Antarctic sea ice extent.

The variations in Antarctic SIE over the past two decades have coincided with a climatological shift toward the positive phase of the SAM (10, 50). The proposed two-timescale surface temperature response to positive SAM is generally attributed to the ocean’s overturning response to circumpolar wind perturbations (13, 27). Our results show that precipitation trends can effectively suppress wind-driven warm water upwelling on multiyear timescales. Thus, a complete theory for how SAM may drive surface temperature and sea ice trends should also account for the impacts of associated hydrological changes on stratification and subsurface heat ventilation.

Last, our findings underscore the importance of sustained autonomous monitoring of hydrographic conditions within the Antarctic SIZ. Year-round in situ measurements are essential for evaluating theories concerning Antarctic sea ice variability and for underpinning confidence in regional climate projections.

## Materials and Methods

### Hydrographic Data.

In situ hydrographic data were retrieved from the Argo Global Data Assembly Center (GDAC) on August 25, 2025. We used the EuroArgo Selection Tool to select all profile data south of 50°S. These data were collected and made freely available by the International Argo Program and the national programs that contribute to it (https://argo.ucsd.edu, https://www.ocean-ops.org). The Argo Program is part of the Global Ocean Observing System.

Argo floats typically drift at a parking depth of 1,000 m and collect conductivity-temperature-depth (CTD) profiles of the upper 2000 m every 7 to 10 d (51). We used delayed-mode, “adjusted” temperature and salinity profiles that include corrections for known biases, such as sensor drift. Only measurements flagged as “good” data (QC-flag = 1) were retained for our analysis. We removed profiles that have a minimum depth deeper than 50 m or a maximum depth less than 1,000 m. The remaining data were linearly interpolated onto a uniform vertical grid with 1 m spacing.

Hydrographic variables were computed from individual profiles before bin-averaging on a $2^{{}^{\circ}}\times 4^{{}^{\circ}}$ latitude-longitude grid for each month between 2005 and 2024. Mixed layer depth (MLD) is defined as the depth where potential density exceeds that of the shallowest measurement by 0.03 kg/m^3^. Potential density is computed using the Gibbs Seawater Toolbox (52). The sub-mixed-layer temperature and salinity gradients are calculated over the 25 m immediately below the MLD. The subsurface potential temperature maximum, $\theta_{\max}$, was identified by first finding the near-surface temperature minimum, $\theta_{\min}$, corresponding to winter mixed layer waters, and then the maximum temperature deeper than $\theta_{\min}$. We further restrict the analysis to the SIZ, empirically defined as regions where the climatological winter mixed layer temperature (June–September average) is below −1 °C.

### Atmospheric Forcing.

Estimates of near-surface winds, precipitation, and evaporation were obtained from the European Centre for Medium-Range Weather Forecasts (ECMWF)’s ERA5 atmospheric reanalysis (53). These fields were provided on a uniform 0.25° spatial grid. The freshwater fluxes were downloaded as monthly averages, while the 10-m wind vectors were downloaded at 6-hourly time steps. The latter was used to compute daily averaged surface ocean stresses, as described below.

### Sea Ice Concentration, Season Length, and Velocity.

Antarctic sea ice concentration (SIC) estimates were obtained using version 5 of the Climate Data Record (CDR), derived from passive microwave satellite data (54). The dataset, provided by the National Snow and Ice Data Center (NSIDC), is available on a 25 × 25 km polar stereographic grid at daily resolution from November 1978 to December 2024. Following ref. 55, sea ice season length was defined as the difference between the dates of sea ice advance and retreat. The annual search window for sea ice season length starts and ends on February 15, the approximate date of minimum Antarctic SIE. In a given grid cell, sea ice advances when that cell is covered by more than 15% SIC for five consecutive days and retreats when SIC decreases below 15% for five days.

Sea ice velocity was obtained from version 4 of the NSIDC Polar Pathfinder 25 km EASE-Grid Sea Ice Motion Vectors product (56), which provides daily fields from 1978 to 2023. For the Antarctic, motion vectors were derived by tracking features in consecutive satellite images. Here, we use velocity measurements since 2000, which roughly overlaps with the available Argo float data.

### Surface Geostrophic Currents.

Surface geostrophic velocities were obtained from the satellite altimetry-based “Gridded and Along-Track Sea Level Heights and Geostrophic Currents” product for the Antarctic region (57, 58), version 2.0, which was processed by SSALTO/DUACS and distributed by AVISO+ with support from CNES. We use the gridded multimission estimates, which are provided as three-day averages from 2013 to 2021 on a 25 km EASE grid.

### Surface Ocean Stress.

Surface ocean stress is computed for the SIZ following the methodology described by refs. 59 and 60. Total surface stress τ is defined as

[1] $$\begin{array}{c} \tau =\alpha \;\tau_{i}+\left(1-\alpha \right)\;\tau_{a}, \end{array}$$

where $\tau_{i}$ and $\tau_{a}$ are the ice–ocean and air–ocean surface stress and α is SIC. The air–ocean stress is given by

[2] $$\begin{array}{c} \tau_{a}=\rho_{a}C_{\mathrm{Da}}\;\left|u_{a}\right|u_{a}, \end{array}$$

where $\rho_{a}=1.25$ kg/m^3^ is the density of air, $C_{\mathrm{Da}}=0.00125$ is the air–sea drag coefficient, and $u_{a}$ is the 10-m wind velocity. Similarly, the ice–ocean stress is defined as

[3] $$\begin{array}{c} \tau_{i}=\rho_{o}C_{\mathrm{Di}}\;\left|u_{\mathrm{rel}}\right|u_{\mathrm{rel}}, \end{array}$$

where $\rho_{o}=1,025$ kg m^−3^ is a reference seawater density, $C_{\mathrm{Di}}=0.0055$ is the ice–ocean drag coefficient, and $u_{\mathrm{rel}}=u_{i}-\left(u_{g}+u_{e}\right)$ is the velocity of sea ice relative to the surface ocean. The surface geostrophic velocity $u_{g}$ was obtained from satellite altimetry (as described above). The surface Ekman velocity is defined by $u_{e}=\tau \sqrt{2}\;e^{i\pi /4}/\left(f\;D_{e}\rho_{o}\right)$, where f is the Coriolis parameter and $D_{e}$ is the Ekman layer depth. We use a latitudinally varying Ekman layer depth defined by $D_{e}=\sqrt{2A_{z}/\left|f\right|}$, where $A_{z}=0.05$ m^2^ s^−1^ is the vertical eddy viscosity. Following ref. 60, τ and $u_{e}$ are computed using a Richardson iteration scheme. Iterations were stopped when consecutive values of $u_{e}$ differed by less than $1\times 10^{-5}$ m s^−1^, which generally occurred within 20 iterations.

All input datasets were bilinearly interpolated to the 25 × 25 km polar stereographic grid that is native to the NSIDC SIC dataset using the xESMF geospatial interpolation Python package (https://xesmf.readthedocs.io/en/stable/index.html). Data were converted to daily resolution by averaging 6-hourly ERA5 winds and linearly upsampling geostrophic velocities. Combining these datasets, we calculated daily averaged τ for 2013–2021, corresponding to the availability of surface geostrophic velocities. We also computed τ using repeating daily averaged $u_{g}$ from 2014, which demonstrated that $u_{g}$ has a relatively small impact on interannual variations in τ. Similarly, we computed τ assuming repeating SIC from 2014 (*SI Appendix*, Fig. S10), which shows that Ekman upwelling after 2016 was slightly amplified by sea ice loss.

### 1D Sea Ice–Ocean Model.

To elucidate the drivers of stratification and sea ice variations in the Weddell Sea, we adapt the 1D modified Price–Weller–Pinkel (PWP; 61) ocean mixed layer model coupled to a sea ice layer, as introduced by ref. 45, which provides an idealized representation of vertical mixing and thermodynamic processes across the Antarctic SIZ. The simplicity of the PWP+ice model precludes a fully accurate representation of observed spatial sea ice variations. However, the goal is to capture relevant leading order processes, specifically the impacts of upwelling and surface freshwater fluxes on interannual sea ice variability. The model’s initialization and numerical configuration are described in *SI Appendix*, Supporting Text; the key physical processes are summarized below.

#### Surface thermodynamics.

The surface heat budget is given by

[4] $$\begin{array}{c} Q_{\mathrm{net}}=\left(1-a\right)Q_{\mathrm{sw}}^{↓}\left(z=0\right)+Q_{\mathrm{lw}}^{↓}-\sigma T_{s}^{4}, \end{array}$$

where $Q_{\mathrm{sw}}^{↓}\left(z=0\right)$ and $Q_{\mathrm{lw}}^{↓}$ are downward shortwave and longwave radiation at the surface, a is the surface albedo, $\sigma =5.67\times 10^{-8}$ W m^−2^ K^−4^ is the Stefan–Boltzmann constant, and $T_{s}$ is the surface temperature in kelvins. The last term in [**4**] represents the surface outgoing longwave radiation. $Q_{\mathrm{lw}}^{↓}$ is applied to the surface layer while $Q_{\mathrm{sw}}^{↓}$ is distributed over the water column with exponentially decaying amplitude, as described by ref. 62. a is equivalent to 0.65 or 0.35 in ice-covered and ice-free conditions, respectively—the model assumes 100% sea ice cover when ice is present. Turbulent heat fluxes are neglected for simplicity since simulating these fluxes would require a prognostic atmospheric layer. Prescribing these fluxes is undesirable since they depend on surface temperature and sea ice conditions, which the model determines. $Q_{\mathrm{net}}$ is applied to the surface layer to evolve $T_{s}$.

If $T_{s}$ is cooled below the freezing point, the excess cooling is converted into an equivalent thickness of sea ice using the latent heat of fusion. We employ the common simplification that sea ice is in thermal equilibrium with the atmosphere (63–65) by requiring that $T_{s}$, here the ice surface temperature, satisfies

[5] $$\begin{array}{c} Q_{\mathrm{net}}=Q_{i}=\kappa_{i}\;\frac{T_{f}-T_{s}}{h_{i}}, \end{array}$$

where $Q_{i}$ is the upward heat flux conducted through sea ice, $\kappa_{i}=$ 2 W m^−1^ °C^−1^ is the thermal conductivity of sea ice, and $T_{f}=-2$ °C is the approximate freezing point of seawater. When sea ice is present, $T_{s}\leq T_{f}$. The thickness of the sea ice layer is governed by the balance of the surface and basal heat fluxes:

[6] $$\begin{array}{c} \frac{dh_{i}}{\mathrm{dt}}=-\frac{Q_{\mathrm{net}}+Q_{\mathrm{oi}}}{L_{i}\;\rho_{i}}, \end{array}$$

where $\frac{dh_{i}}{\mathrm{dt}}$ is the basal growth rate, $Q_{\mathrm{oi}}$ is the ocean-ice basal heat flux, $L_{i}=3.3\times 10^{5}$ J/kg and $\rho_{i}=920$ kg/m^3^ are the latent heat of fusion and density of sea ice, respectively. Following ref. 66, the basal ice heat flux is given by

[7] $$\begin{array}{c} Q_{\mathrm{oi}}=\rho_{o}\;c_{p}\;C_{\mathrm{Di}}\;u^{*}\;\left(T_{\mathrm{ml}}-T_{f}\right), \end{array}$$

where $c_{p}=4,000$ J kg^−1^ °C^−1^ is the specific heat capacity of seawater, $T_{\mathrm{ml}}$ is the mixed layer temperature, and $u^{*}=\sqrt{\tau /\rho_{o}}$ is the friction velocity.

#### Surface freshwater budget.

The model is forced by prescribed surface freshwater fluxes, specifically precipitation P and evaporation E. Additionally, a salinity flux is introduced during sea ice growth or melt. These processes evolve the salinity of the ocean surface layer $S_{s}$ according to

[8] $$\begin{array}{c} \frac{dS_{s}}{\mathrm{dt}}=\frac{1}{\delta z}\left[\left(E-P\right)\;S_{\mathrm{ref}}+\left(S_{\mathrm{ref}}-S_{i}\right)\;\frac{dh_{i}}{\mathrm{dt}}\right], \end{array}$$

where δz is the thickness of the model’s top layer, $S_{\mathrm{ref}}=34$ PSU is a reference salinity for seawater, and $S_{i}=5$ PSU is the salinity of sea ice.

#### Vertical mixing and advection.

The PWP model parameterizes vertical mixing that results from static and shear instabilities (61). These instabilities are resolved by progressively deepening the mixed layer until stability is restored. Shear instability is evaluated using a bulk Richardson number criterion. Additionally, the model simulates sub-mixed-layer upwelling and vertical mixing processes using a simple advection–diffusion balance, shown here for temperature:

[9] $$\begin{array}{c} \frac{\mathrm{dT}}{\mathrm{dt}}=w_{\mathrm{ek}}\;\frac{\mathrm{dT}}{\mathrm{dz}}+\kappa_{z}\;\frac{d^{2}T}{dz^{2}}, \end{array}$$

where $w_{\mathrm{ek}}=\nabla \times \left(\frac{\tau }{\rho_{o}f}\right)\cdot \hat{z}$ is the rate of Ekman upwelling and $\kappa_{z}=$$5\times 10^{-5}$ m^2^ s^−1^ is a vertical turbulent diffusion coefficient typical for the Weddell Sea (67). Salinity is evolved using an identical formulation. To solve Eq. **9**, we use a second-order finite differencing scheme, holding the properties of the bottom grid cell fixed. $w_{\mathrm{ek}}$ is computed using the extended record of τ assuming repeating ocean surface geostrophic currents from 2014 (Fig. 4*B*).

## Supplementary Material

Appendix 01 (PDF)

## Data Availability

The hydrographic, sea ice, and atmospheric reanalysis data used in this study were all sourced from publicly available repositories described above. The Python code used to process the data and generate the figures is available on Zenodo (https://doi.org/10.5281/zenodo.17460241).
