# Supplementary material for: Recent extremes in Antarctic sea ice extent modulated by ocean heat ventilation
Source: Proc Natl Acad Sci U S A. 2026 Mar 23;123(14):e2530832123. doi: 10.1073/pnas.2530832123 (PMC13056085; doi:10.1073/pnas.2530832123)
Supplement: Supplementary file 1 — Appendix 01 (PDF) [file pnas.2530832123.sapp.pdf]

PNAS

Supporting Information for

Recent extremes in Antarctic sea ice extent modulated by ocean heat ventilation

Earle A. Wilson, Lexi Arlen and Ethan C. Campbell

Corresponding author: Earle Wilson
E-mail: earlew@stanford.edu

This PDF file includes:

Supporting text
Figs. S1 to S17
SI References

Supporting Information Text

Methods

1D ice–ocean model configuration. We adapt the 1D modified Price–Weller–Pinkel (PWP; 1) ocean mixed layer model coupled to a sea ice layer, as introduced by (2). The relevant physical processes are described in the *Materials and Methods* of the main text. Additional details about the model's configuration are provided below.

Model initialization and forcing. The model was initialized with upper 250 m of temperature and salinity profiles from the Weddell Sea SIZ (Fig. S13). We used 40 randomly selected summertime Argo profiles collected in the western Weddell Sea (62°S–70°S and 40°W–70°W) between years 2005–2008. This time period corresponds to the first three years of consistent Argo float measurements in the Weddell Sea and provide the best estimates of upper ocean conditions prior to record-breaking sea ice expansion and retreat over the following two decades (3, 4). Since the model simulations begin on January 1, the summertime profiles were restricted to those collected between December 15 and January 15. Each profile was linearly interpolated to a vertical grid with 2-m spacing. The model was forced with precipitation minus evaporation (P-E), downward shortwave ($Q^{\downarrow}_{\mathrm{sw}}$) and longwave ($Q^{\downarrow}_{\mathrm{lw}}$) radiative fluxes, surface ocean stress (τ), and Ekman upwelling ($w_{\mathrm{ek}} \propto [\nabla \times \tau] \cdot \hat{z}$). Each forcing is linearly interpolated to 6-hourly resolution, the model's time step.

Control and perturbation experiments. The control simulation was evolved using annually repeating surface forcing from the year 2000, which was equilibrated over 60 years (Figure S14, S15). The equilibrated simulations approximately reproduce the observed seasonality of upper ocean and near-surface properties, with wintertime mixed layer depth and sea ice thickness reaching ∼120 m and 0.8–1 m, respectively. Three perturbation simulations were branched from the equilibrated control run, whereby each is individually forced with observed interannually varying Ekman upwelling w_{ek} (estimated from surface stresses assuming geostrophic velocities from 2014; Fig. S10), $P - E$, and the combination of both forcings for the 2000–2023 period. Each perturbation was repeated with 40 initial profiles for a total of 160 simulations, including the control run.

Model equilibration and stability. For simplicity, these simulations neglect lateral processes, such as ocean and sea ice advection and turbulent air-sea heat fluxes. However, initial testing revealed that imposing freshwater fluxes from only $P - E$ resulted in some ensemble members having unreasonably weak stratification. These profiles become completely convectively unstable in the winter, which often results in the total melting of the sea ice layer. Though deep convection and wintertime open-ocean polynyas can occur in the Weddell Sea (5, 6), such events are rarely observed. To ensure that the model equilibrates to a realistic base state, we introduce a constant surface freshwater flux (F_{adv}) of 5 cm per year to help stabilize the water column. This additional freshwater flux is modest compared to the net freshwater flux associated with sea ice advection, which is estimated to range between 0–0.5 m per year within the off-shelf regions of the Weddell Sea, with the lower end of this range being more typical for our focus region (7, 8). Since interannually varying freshwater fluxes from sea ice advection remain poorly constrained by observations, we treat this quantity as a tuning parameter. Doubling F_{adv} to 10 cm per year does not qualitatively change our main results.

The model uses the standard convective adjustment and bulk Richardson number mixing schemes to resolve static and shear instabilities, respectively (1). For computational efficiency, we do not employ the gradient Richardson number mixing scheme, which evaluates and iteratively resolves shear instabilities between each vertical grid cell. Limited testing reveals that this algorithm has negligible impact on our simulations. Additionally, upper ocean velocities are relaxed to initial conditions (zero velocity) over a 7-day time scale to prevent a long-term drift in velocities.

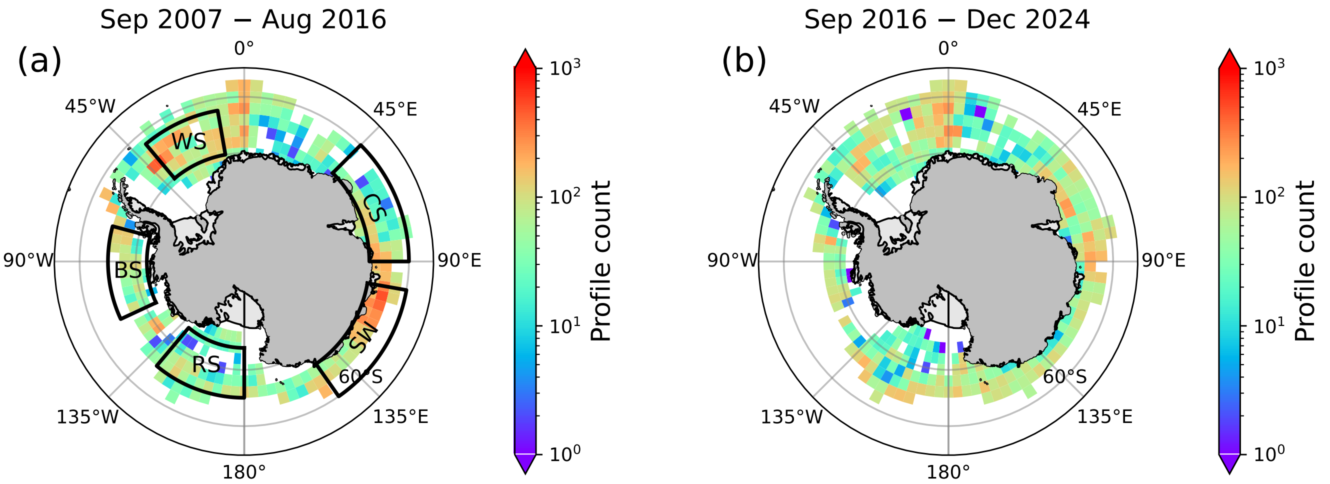

Fig. S1. Total number of Argo profiles for (a) the sea ice expansion period (September 2007–August 2016) and (b) the sea ice retreat period (September 2016–December 2024). Argo profile data have been aggregated into $4^\circ \times 2^\circ$ longitude-latitude bins and displayed for the Antarctic seasonal sea ice zone (SIZ), empirically defined as regions where the average winter (June–August) mixed layer temperature (MLT) is cooler than -1° C. The displayed sums reflect the subset of profiles that meet the quality control checks described in the *Materials and Methods* of the main manuscript. The sea ice growth and retreat periods are based on the statistical change point analysis described by (3). The boxes outline the western Weddell Sea (WS), referenced in the main text, as well as the Cooperation, Mawson, Ross and Bellingshausen Sea regions, referenced by Figs. S5–S9 respectively.

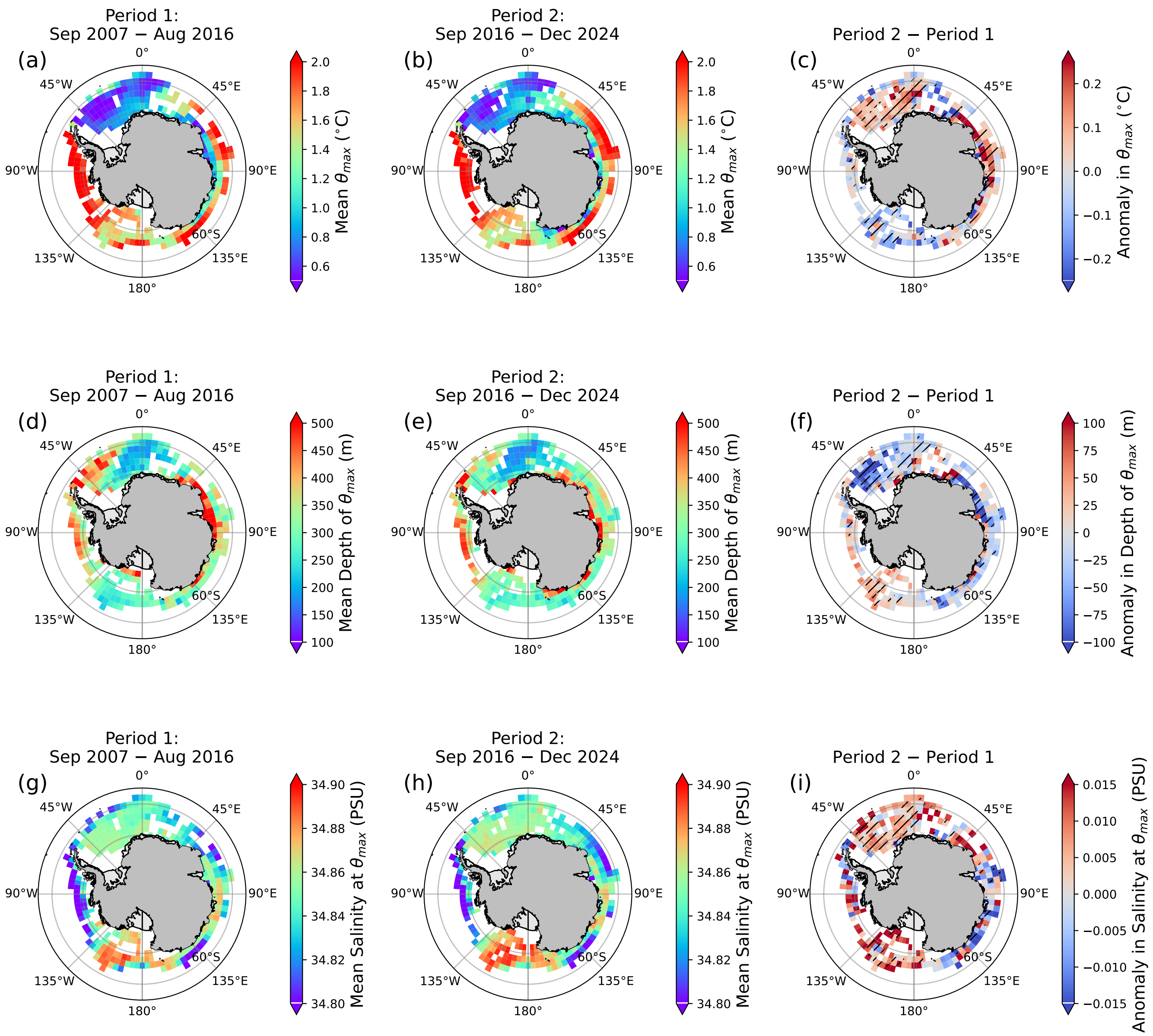

Fig. S2. The spatial distribution of subsurface potential temperature maximum $\theta_{\max}$ in the Antarctic SIZ. (a,b) Mean $\theta_{\max}$ for the Antarctic sea ice expansion and retreat periods, as defined in Fig. S1, and (c) their difference. Middle (d-f) and bottom (g-i) rows: like (a-c) but for the depth of $\theta_{\max}$ and the salinity at the depth of $\theta_{\max}$, respectively. In (c), (f), and (i), the hatched regions denote grid cells where the differences are statistically significant at the 95% confidence level, based on 1,000 bootstrap resamples of the monthly-averaged data. Note that panels (c) and (f) are identical to Figure 2 in the main text. However, the density of the hash markings vary based on the figure size.

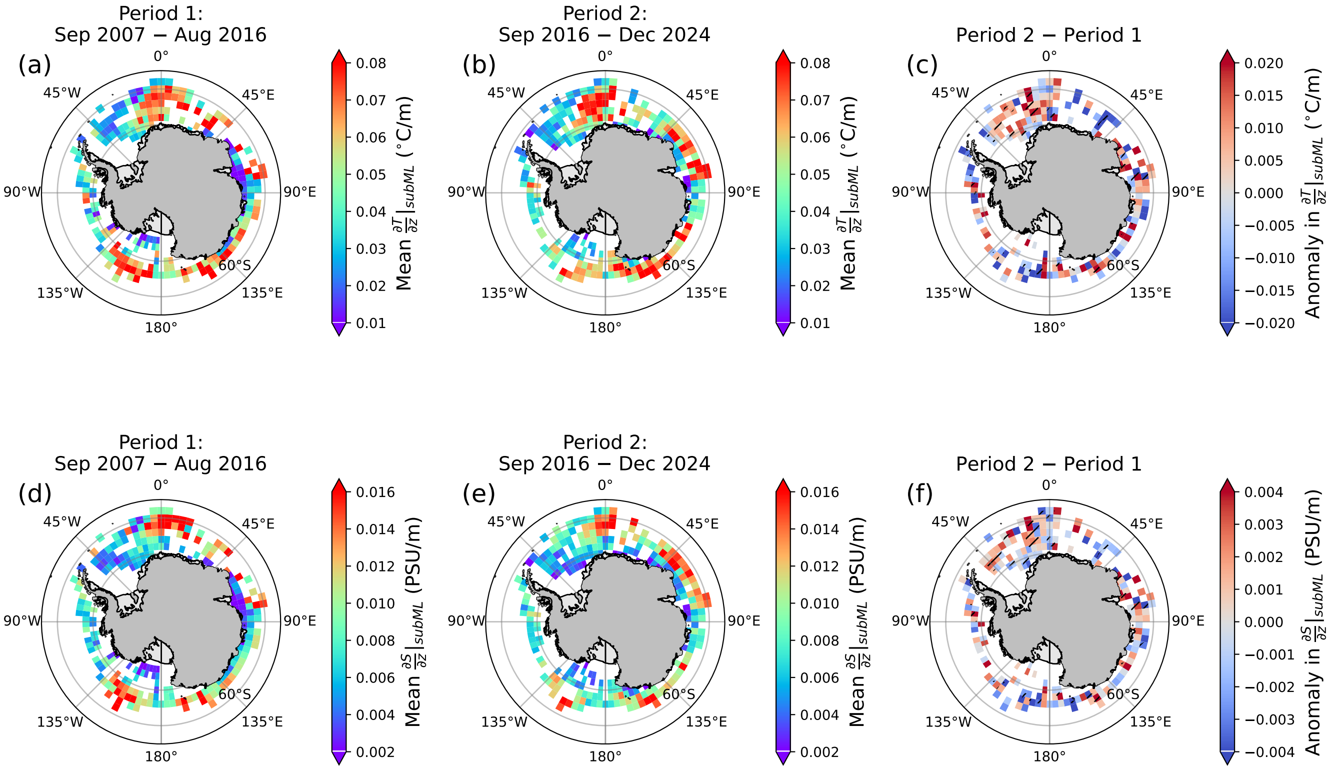

Fig. S3. The spatial distribution of wintertime (June-September) sub-mixed layer temperature and salinity gradients in the Antarctic SIZ. (a,b) Mean sub-mixed layer temperature gradient for the Antarctic sea ice expansion and retreat periods, as defined in Fig. S1, and (c) their difference. Bottom row (d-f): like (a-c) but for the salinity gradient. Gradients are computed by linearly regressing measurements 25 m below the mixed layer. Hash markings in (c) and (f) are the same as in Fig. S2.

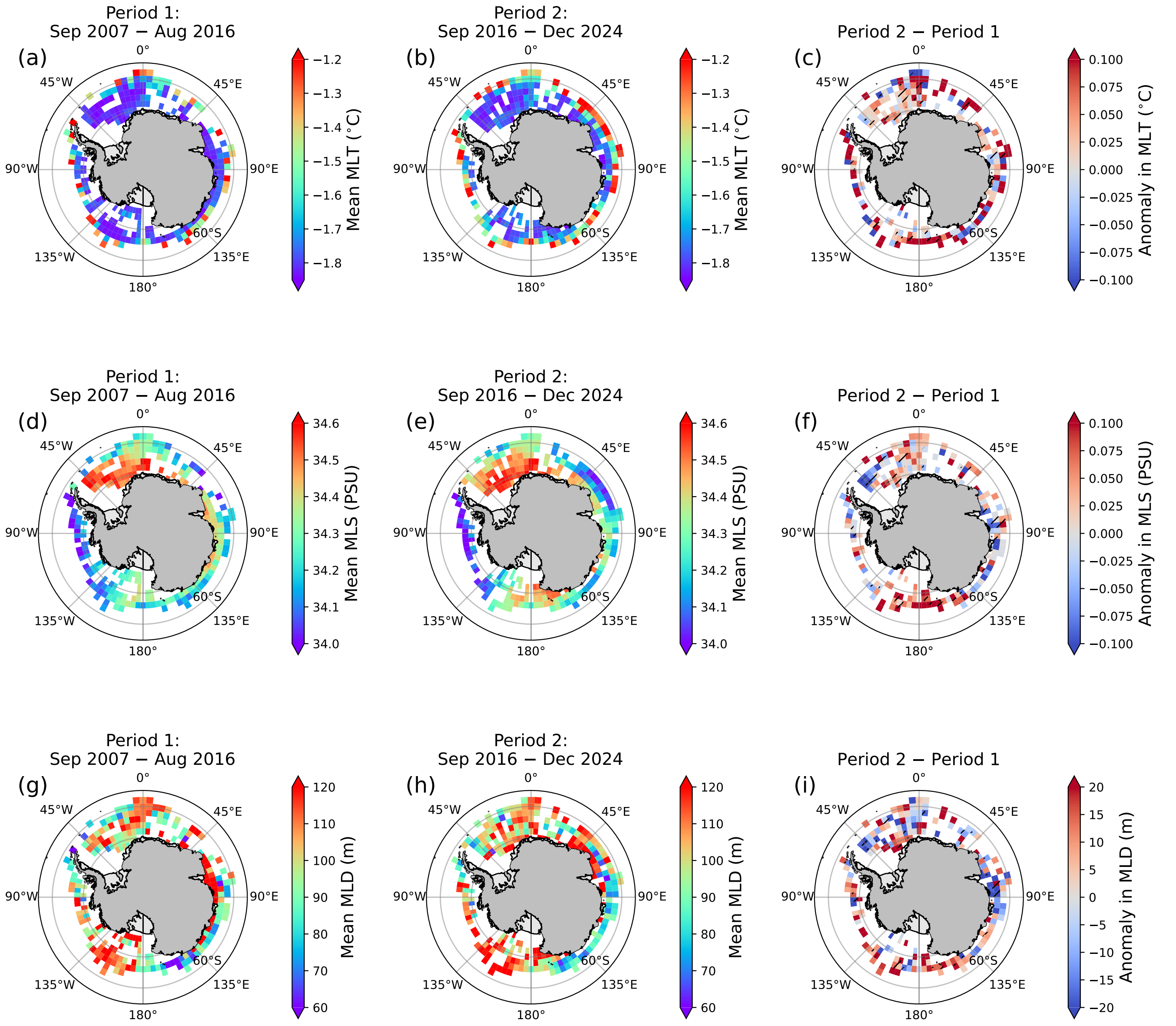

Fig. S4. The spatial distribution of wintertime (June-September) mixed layer temperature (MLT), salinity (MLS) and depth (MLD) in the Antarctic SIZ. (a,b) Mean MLT for the Antarctic sea ice expansion and retreat periods, as defined in Fig. S1, and (c) their difference. Middle (d-f) and bottom (g-i) rows: like (a-c) but for MLS and MLD, respectively. Hash markings in (c), (f), and (i) are the same as in Fig. S2.

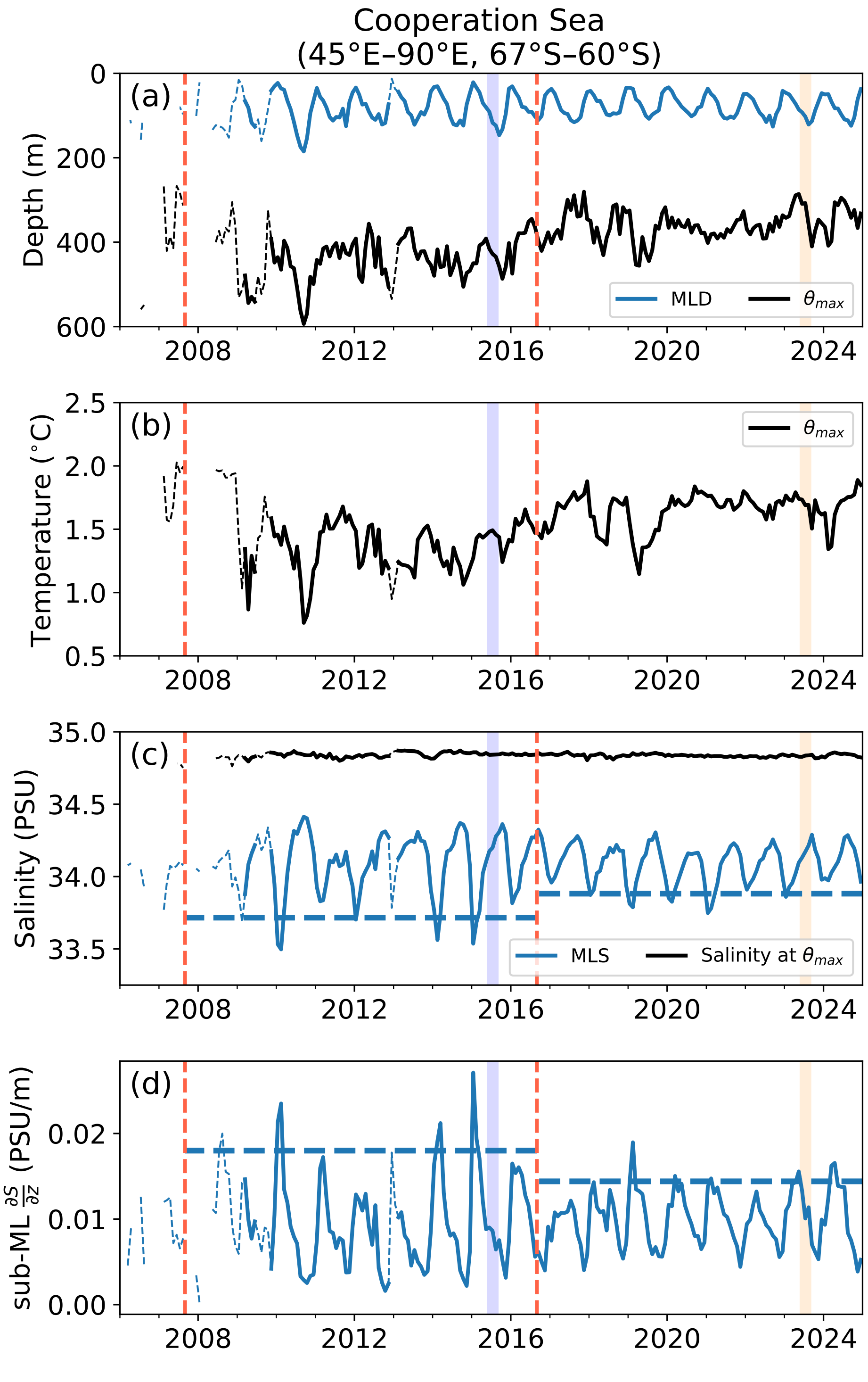

Fig. S5. Monthly averaged upper-ocean properties in the Cooperation Sea, off East Antarctica (see box labeled as "CS" in Fig. S1a). (a) Mixed layer depth (MLD) and depth of $\theta_{\max}$. (b) $\theta_{\max}$. (c) Mixed layer salinity (MLS) and salinity at the depth of $\theta_{\max}$. (d) Salinity gradient below the mixed layer (ML). In panel (c), the dashed horizontal lines show the average annual minimum salinity over the sea ice advance and retreat periods; in panel (d), the horizontal lines represent the average annual maximum vertical salinity gradient for each period. Thin dashed curves represent monthly averages using all available data while thick solid curves highlight months with more than 10 profiles. Vertical lines demarcate the starts of the sea ice advance and retreat periods and the winter SIA maximum and minimum, as in Fig. S1.

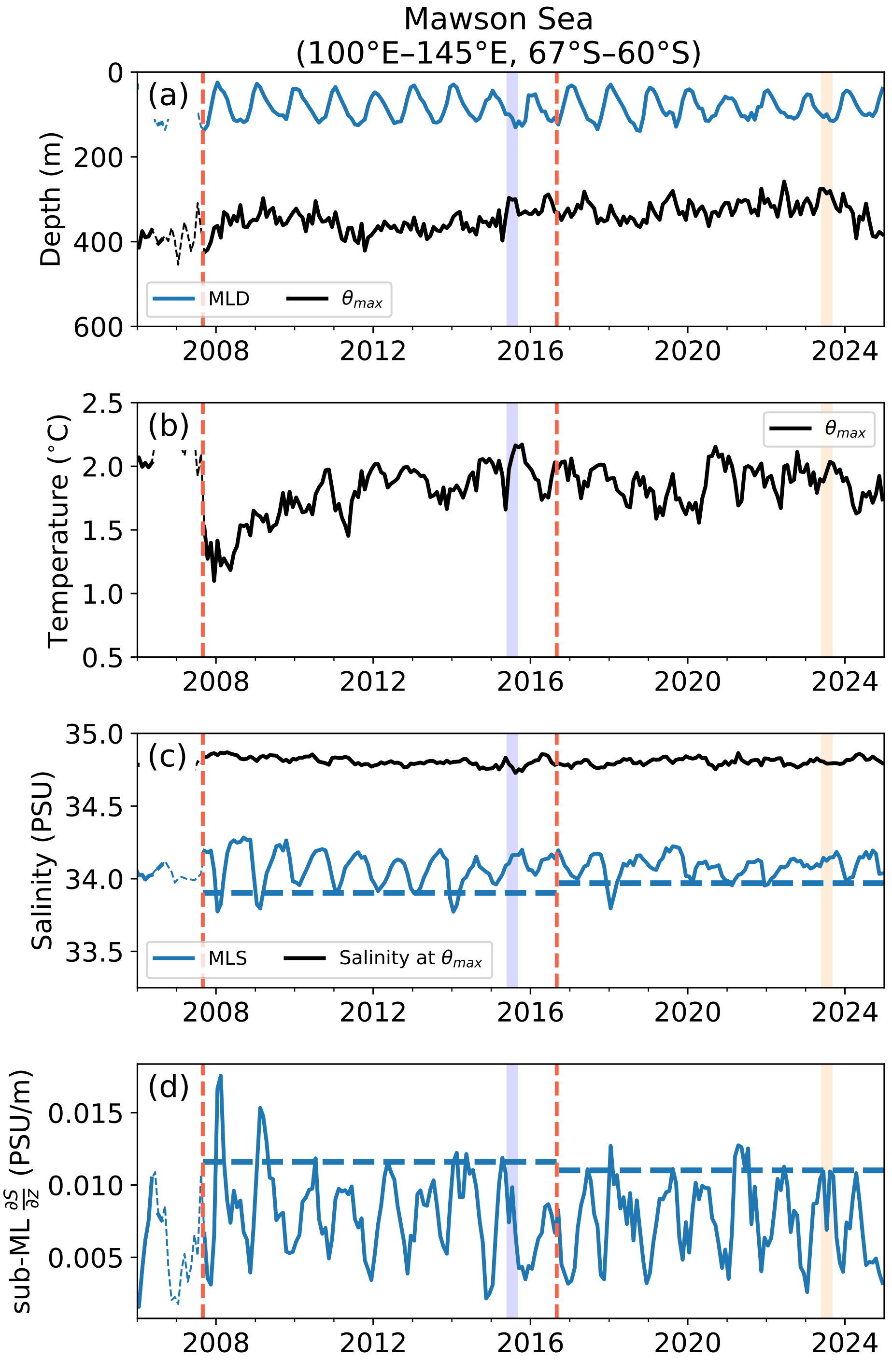

Fig. S6. Like Fig. S5 but for the Mawson Sea, off East Antarctica (see box labeled as "MS" in Fig. S1a).

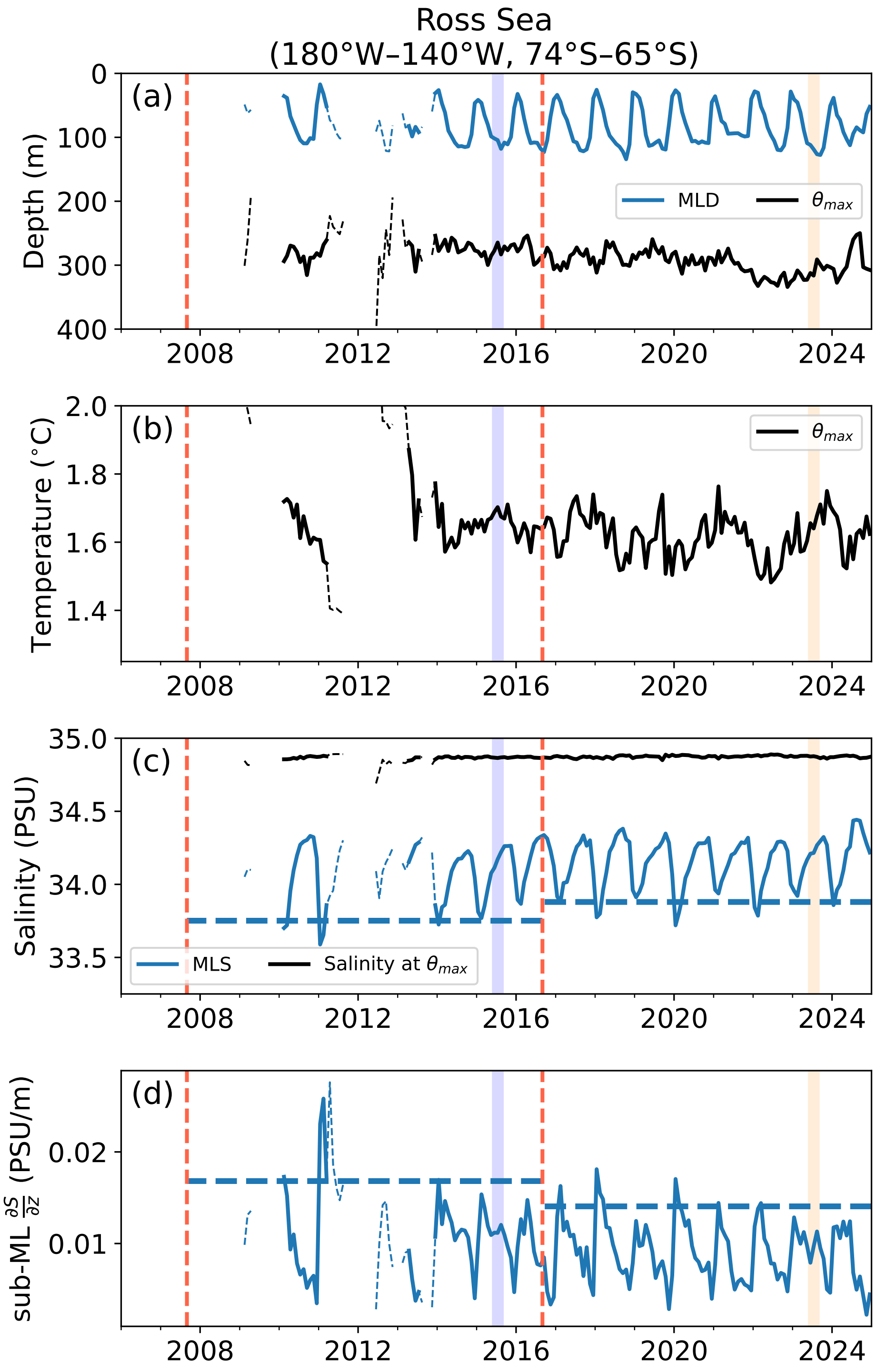

Fig. S7. Like Fig. S5 but for the Ross Sea (see box labeled as "RS" in Fig. S1a).

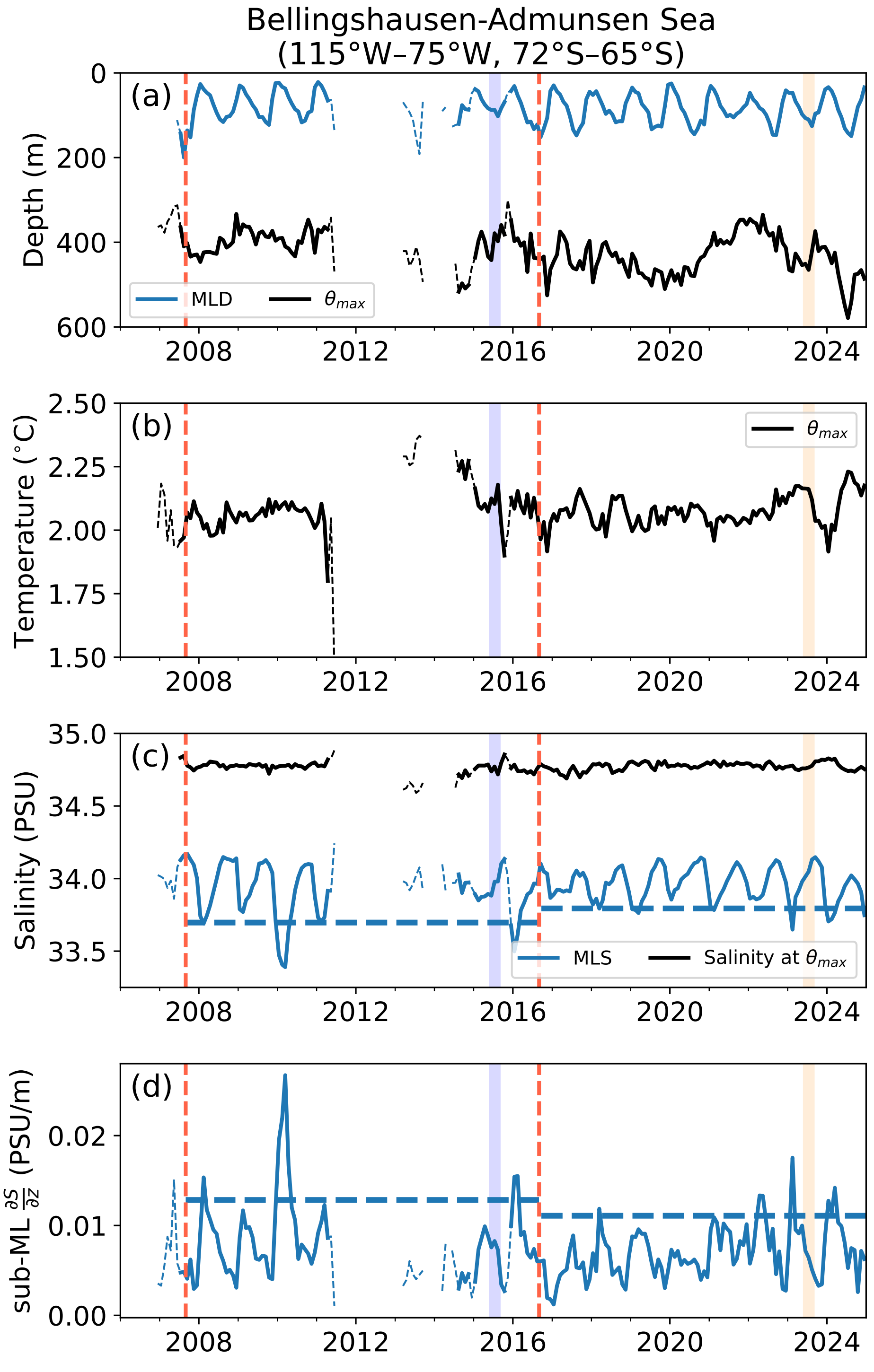

Fig. S8. Like Fig. S5 but for the Bellingshausen Sea (see box labeled as "BS" in Fig. S1a).

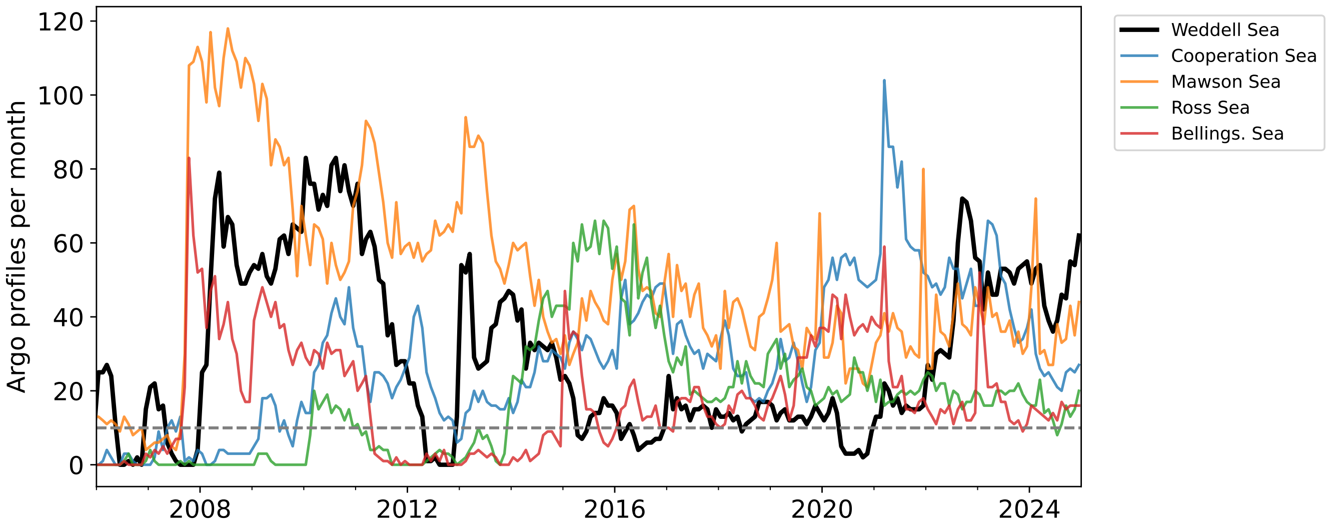

Fig. S9. Time series of available Argo float profiles for the Weddell, Cooperation, Mawson, Ross and Bellingshausen Seas, as defined by boxes in Fig. S1. For reference, the dashed gray line delineates months where there are more than 10 profiles (typically signifying at least three Argo floats) in a given region.

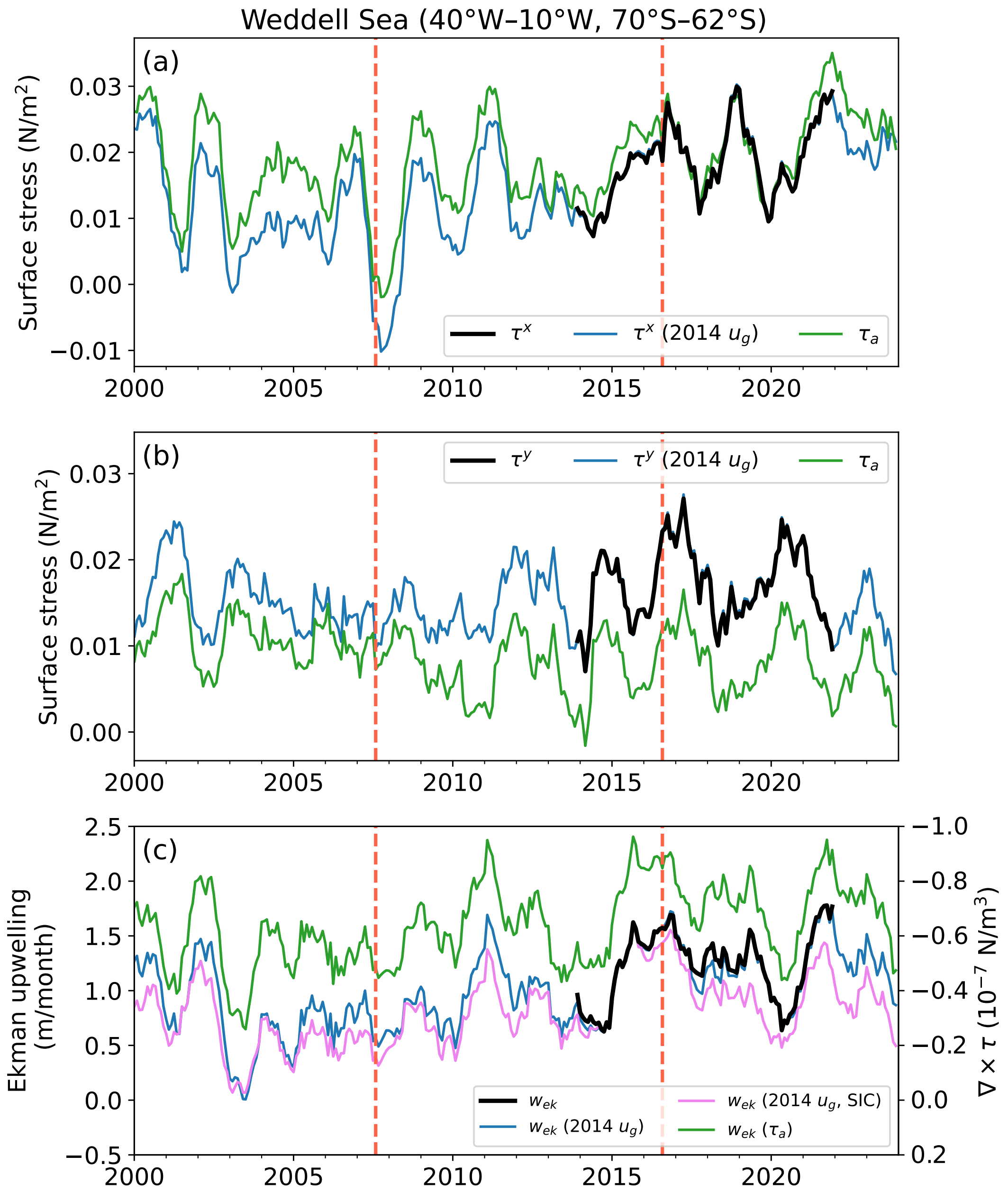

Fig. S10. Monthly averaged surface ocean stress τ from 1988–2023 for the western Weddell Sea, defined by the box in Fig. S1a. (a, b) The zonal and meridional components of the total surface stress, τ^x and τ^y, respectively. (c) Ekman upwelling rates and, equivalently, surface stress curl. Black curves represent values computed using interannually varying surface ocean geostrophic currents $\mathbf{u_g}$, derived from satellite dynamic ocean topography available for 2013–2021 (*Materials and Methods*). Blue curves represent τ using repeating $\mathbf{u_g}$ from 2014. Green curves represent estimates associated with only the air-sea component of the surface ocean stress $\tau_\mathbf{a}$. In (c), the magenta curve represents τ using both repeating $\mathbf{u_g}$ and sea ice concentration (SIC) values from 2014. Red dashed vertical lines outline the starts of the Antarctic sea ice expansion and retreat periods, September 2007 and September 2016, respectively, as defined by (3).

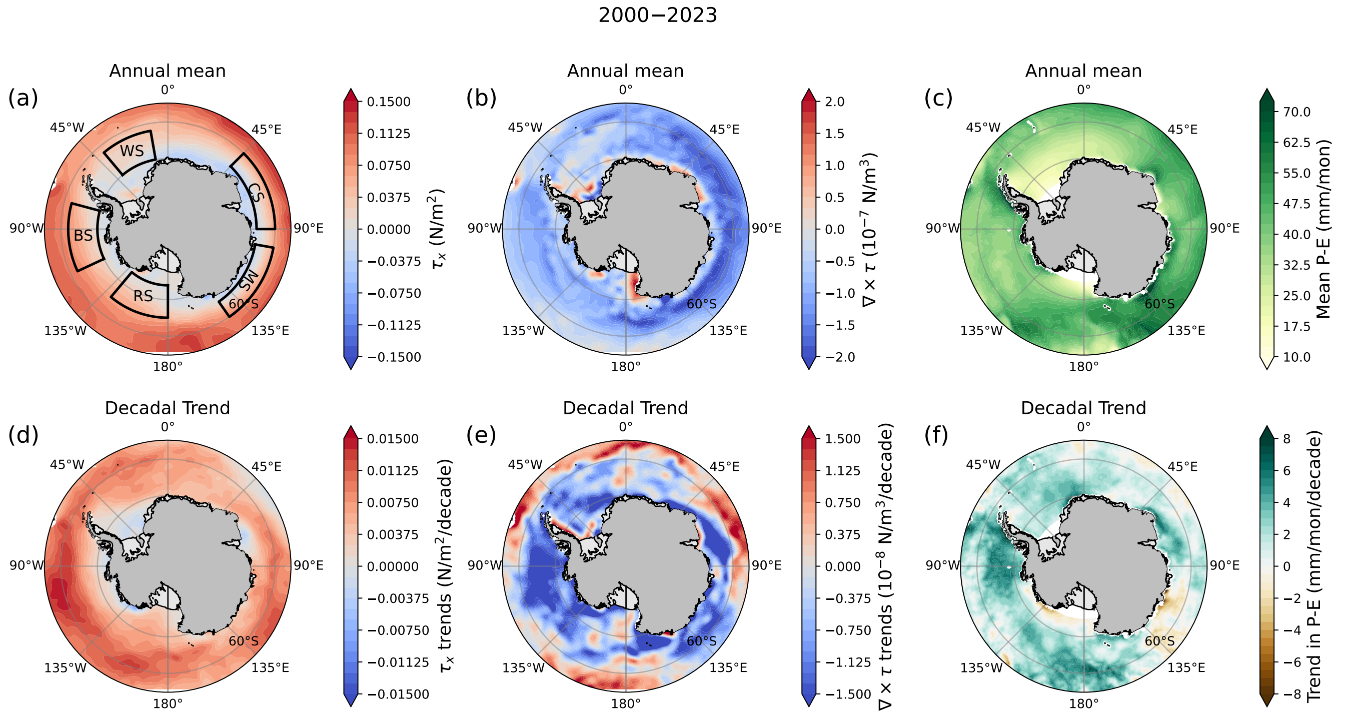

Fig. S11. Annual mean (top row) and decadal trends (bottom row) for zonal surface stress (left column), surface stress curl (middle column), and precipitation minus evaporation (right column) for 2000–2023. In (c) and (f), surface stress is computed using the repeating geostrophic velocities from 2014. The boxes in panel (a) outline the five regions assessed in Fig. S12: western Weddell Sea (WS), Cooperation Sea (CS), Mawson Sea (MS), Ross Sea (RS), and the Bellingshausen-Amundsen Seas (BS).

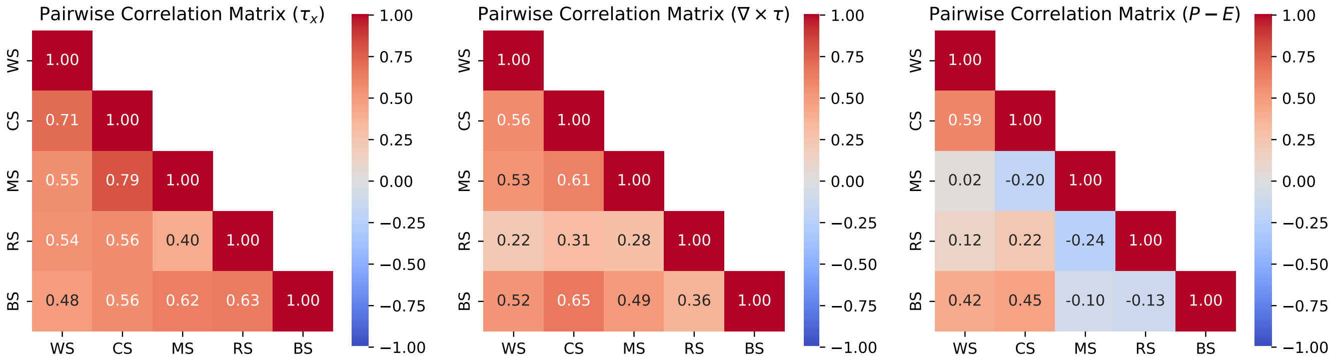

Fig. S12. Pairwise correlation matrices of interannual variations in surface stress, surface stress curl, and net precipitation for the five regions outlined in Fig. S11a. Correlations were computed after smoothing each regional monthly time series with a 12-month running mean.

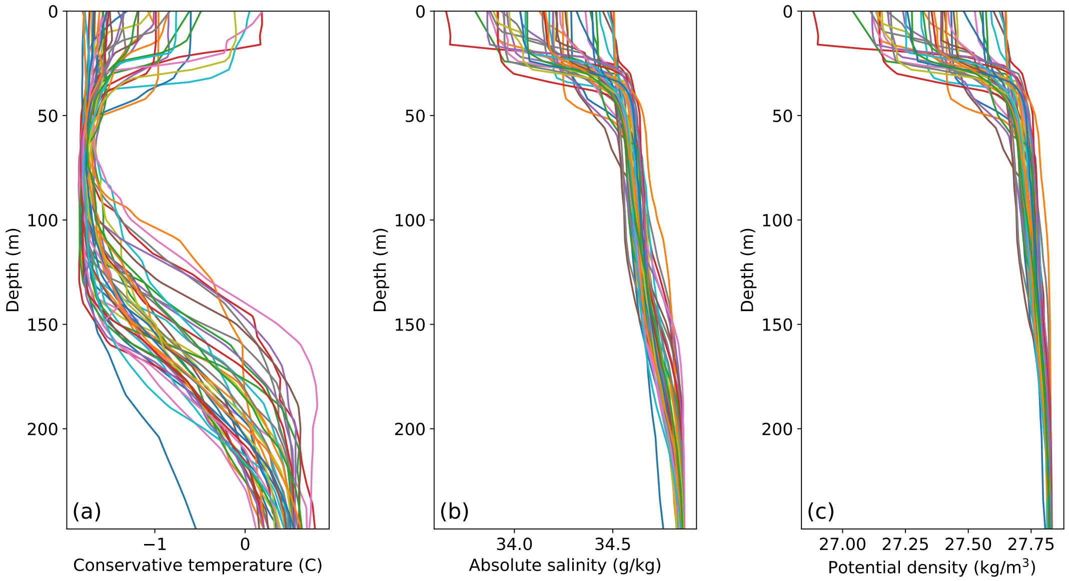

Fig. S13. Summertime Argo float profiles used to initialize the 1D ice–ocean model (*Materials and Methods*). (a-c) Conservative temperature, absolute salinity, and potential density, respectively. A total of 40 hydrographic profiles were randomly selected from those collected in the western Weddell Sea (within 40°W–10°W and 62°S–70°S) between December 15 and January 15.

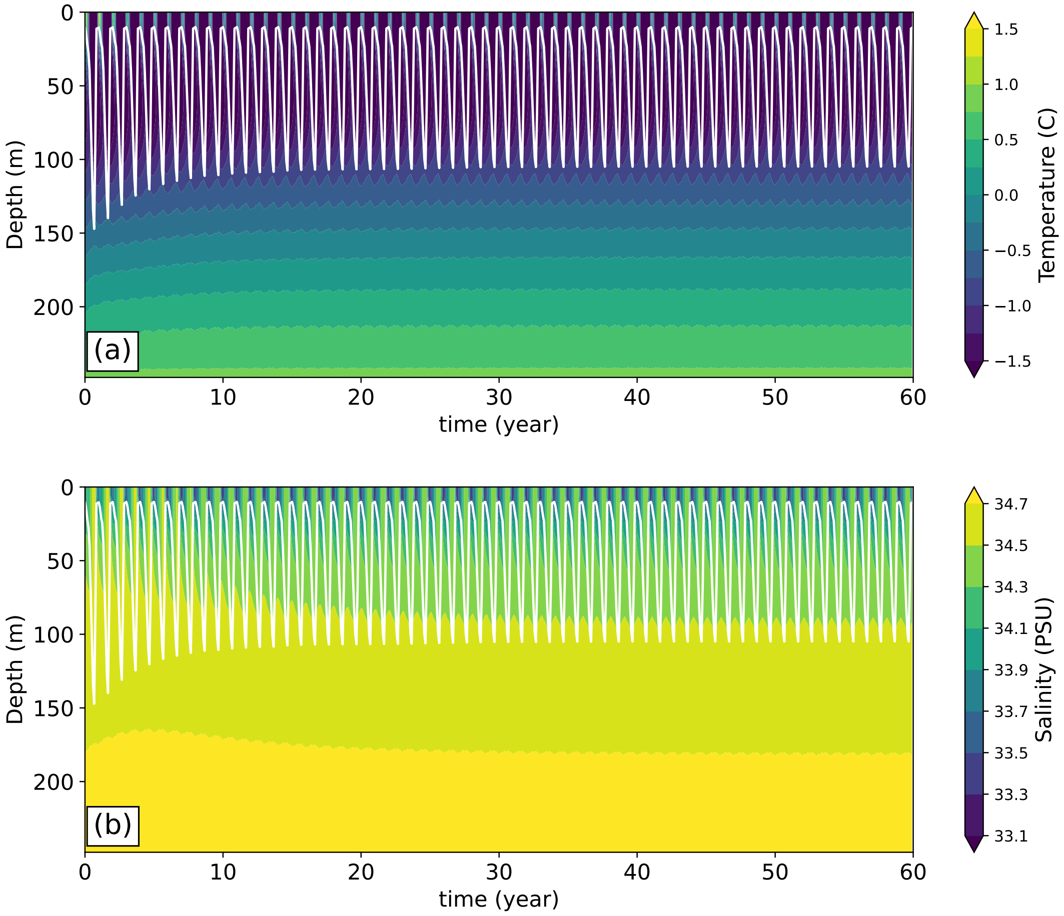

Fig. S14. Example evolution of upper 250 m temperature (a) and salinity (b) simulated by the 1D ice–ocean model during the 60-year control run spin-up (*Materials and Methods*). Here, the model is forced with repeating surface conditions from the year 2000. White lines represent simulated mixed layer depths, defined using a 0.01 kg/m^3 density criterion.

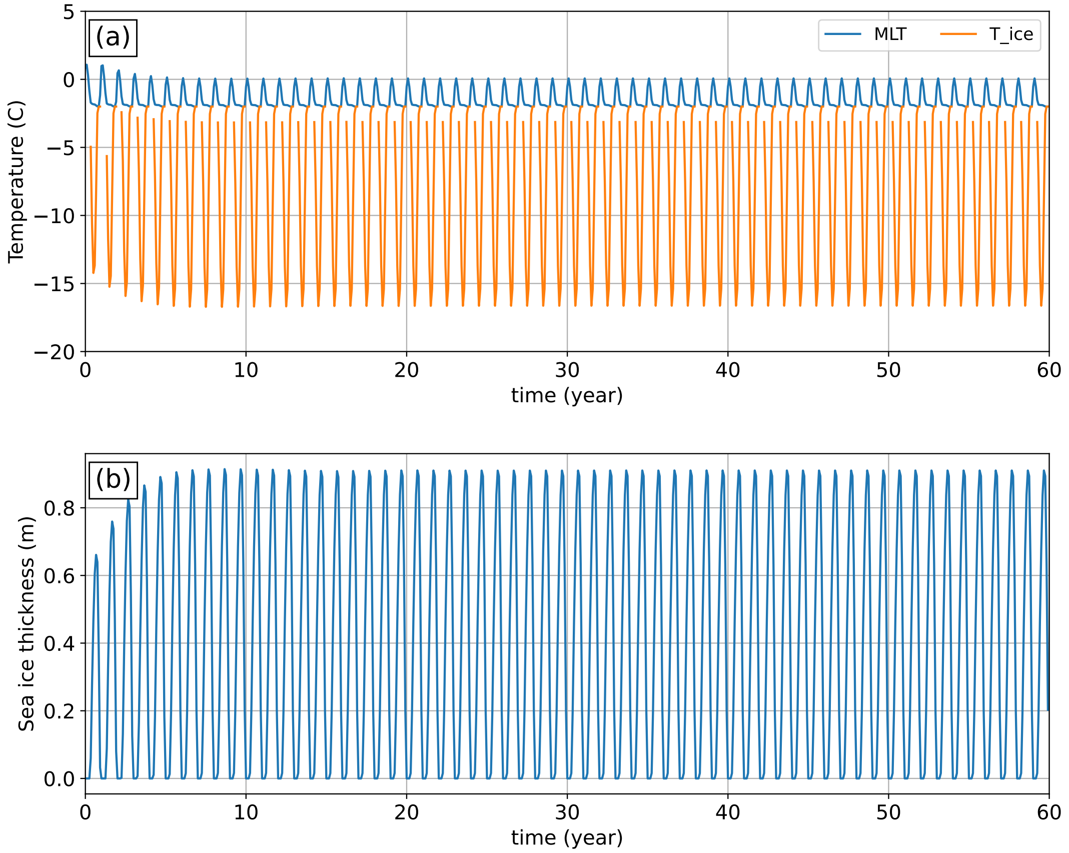

Fig. S15. Like Fig. S14 but showing (a) sea ice surface and ocean mixed layer temperatures and (b) sea ice thickness for the 60-year control run spin-up.

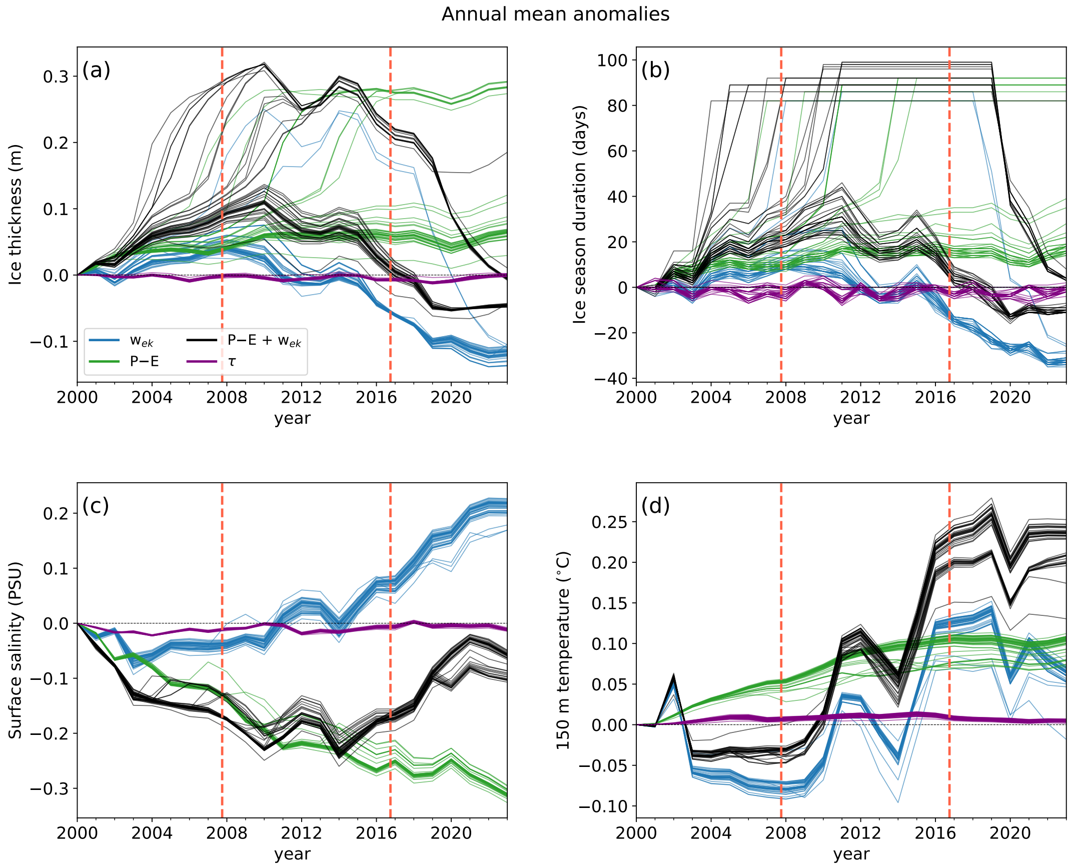

Fig. S16. 1D ice–ocean model simulations of upper ocean and sea ice properties forced with observed Ekman upwelling $w_{\rm ek}$, net precipitation $P-E$, and total surface stress τ across the western Weddell Sea (within 40°W–10°W and 62°S–70°S) between 2000–2023. (a) Annually averaged sea ice thickness anomalies for simulations forced individually with interannually varying $w_{\rm ek}$ (blue), $P-E$ (green), $w_{\rm ek}+P-E$ (black), and τ (purple). Individual lines correspond to simulations initialized with one of the 40 Argo float profiles shown in Fig. S13. Anomalies are relative to control runs equilibrated to surface forcing from 2000. (b-d) Like (a) but showing anomalies in sea ice season duration, ocean surface salinity, and temperature at 150 m depth. In (b), simulations where the ice duration anomalies plateau between 80–100 days correspond to years where sea ice reaches the maximum possible duration of 365 days. The τ perturbation experiments use surface stresses computed using repeated surface geostrophic velocities from 2014 (Fig. S10). Red dashed vertical lines outline the starts of the Antarctic sea ice expansion and retreat periods, September 2007 and September 2016, respectively, as defined by (3).

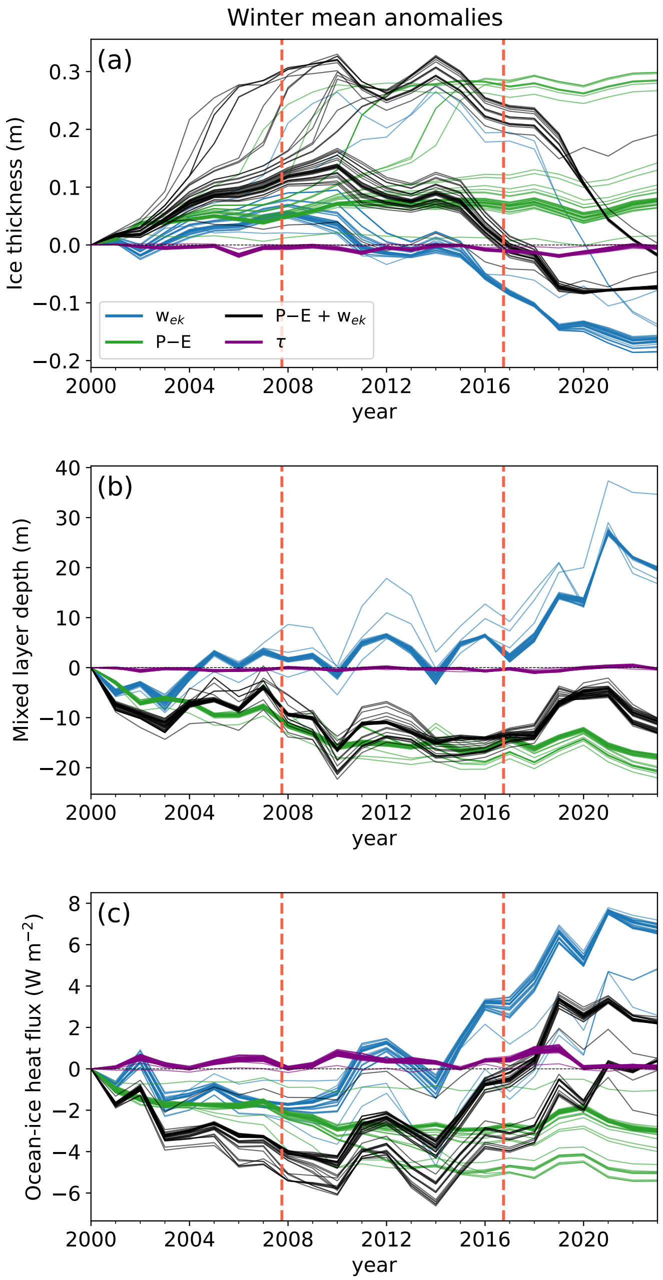

Fig. S17. Like Fig. S16, but showing anomalies in mean wintertime (June-September) sea ice thickness (a), mixed layer depth (b), and ocean-ice heat fluxes (c). Anomalies are relative to the control simulation (year 2000), in which the winter-mean sea ice thickness, mixed layer depth, and ocean-ice heat flux are approximately 0.8 m, 90 m, and 15 W m^{-2}, respectively.

References

1. JF Price, RA Weller, R Pinkel, Diurnal Cycling: observations and models of the upper ocean response to diurnal heating, cooling, and wind mixing. *J. Geophys. Res.* **91**, 8411 (1986).
2. EA Wilson, SC Riser, EC Campbell, AP Wong, Winter upper-ocean stability and ice-ocean feedbacks in the sea ice-covered Southern Ocean. *J. Phys. Ocean.* **49**, 1099–1117 (2019).
3. A Purich, EW Doddridge, Record low Antarctic sea ice coverage indicates a new sea ice state. *Commun. Earth & Environ.* **4**, 314 (2023).
4. C Eayrs, X Li, MN Raphael, DM Holland, Rapid decline in Antarctic sea ice in recent years hints at future change. *Nat. Geosci.* **14**, 460–464 (2021).
5. EC Campbell, et al., Antarctic offshore polynyas linked to Southern Hemisphere climate anomalies. *Nature* **570**, 319–325 (2019).
6. WG Cheon, AL Gordon, Open-ocean polynyas and deep convection in the Southern Ocean. *Sci. Rep.* **9**, 6935 (2019).
7. F Alexander Haumann, N Gruber, M Münnich, I Frenger, S Kern, Sea-ice transport driving Southern Ocean salinity and its recent trends. *Nature* **537**, 89–92 (2016).
8. RP Abernathey, et al., Water-mass transformation by sea ice in the upper branch of the Southern Ocean overturning. *Nat. Geosci.* **9**, 596–601 (2016).
